# Sulfenamide and Sulfonamide Derivatives of Metformin – A New Option to Improve Endothelial Function and Plasma Haemostasis

**DOI:** 10.1038/s41598-019-43083-z

**Published:** 2019-04-25

**Authors:** Magdalena Markowicz-Piasecka, Kristiina M. Huttunen, Marlena Broncel, Joanna Sikora

**Affiliations:** 1 Laboratory of Bioanalysis, Department of Pharmaceutical Chemistry, Drug Analysis and Radiopharmacy, Medical University of Lodz, ul. Muszyńskiego1, 90-151 Lodz, Poland; 2 School Of Pharmacy, Faculty of Health Sciences, University of Eastern Finland, Yliopistonranta 1C, POB 1627, 70211 Kuopio, Finland; 3 Department of Internal Diseases and Clinical Pharmacology, Medical University of Lodz, Kniaziewicza 1/5, 91-347 Lodz, Poland

**Keywords:** Pharmacodynamics, Drug development

## Abstract

Type 2 diabetes mellitus (T2DM) is a multi-factorial disease which can cause multiple organ dysfunction, including that of the vascular endothelium. The aim of the present study was to evaluate the effects of metformin, and its sulfenamide and sulfonamide derivatives (compounds **1–8**) on the selected markers of endothelial function and blood coagulation. The integrity of endothelial cells(ECs) was examined using the real-time cell electric impedance system. Tissue Factor(TF) production, the release of von Willebrand Factor (vWF) and tissue plasminogen activator(t-PA) from ECs were determined using immunoenzymatic assays, while the process of platelet thrombus formation using the Total Thrombus-Formation Analysis System. Sulfenamide with *n*-butyl alkyl chain(**3**) does not interfere with ECs integrity, and viability (nCI_(24h)_ = 1.03 ± 0.03 vs. 1.06 ± 0.11 for control), but possesses anticoagulation properties manifested by prolonged platelet-dependent thrombus formation (Occlusion Time 370.3 ± 77.0 s vs. 286.7 ± 65.5 s for control) in semi-physiological conditions. Both *p*- and *o*-nitro-benzenesulfonamides (compounds**7,8**) exhibit anti-coagulant properties demonstrated by decreased vWF release and prolonged parameters of platelet thrombus formation and total blood thrombogenicity. In conclusion, chemical modification of metformin scaffold into sulfenamides or sulfonamides might be regarded as a good starting point for the design and synthesis of novel biguanide-based compounds with anticoagulant properties and valuable features regarding endothelial function.

## Introduction

Metformin is a commonly used oral drug for the treatment of type 2 diabetes mellitus (T2DM)^1^. Many studies have reported a favourable benefit:risk ratio for metformin^2^ which, together with cost-effectiveness, has resulted in it being included as first-line glucose-lowering therapy for T2DM subjects in international treatment guidelines and algorithms. Glucose-lowering activity of metformin is based on several mechanisms, including inhibition of gastrointestinal absorption of glucose, amelioration of hyperinsulinemia due to increased insulin sensitivity, inhibition of hepatic gluconeogenesis, and increase in tissues’ glucose consumption^3^. In addition, metformin might also improve insulin resistance which has found clinical application in the treatment of polycystic ovary syndrome (PCOS)^3^. Apart from its glucose-lowering properties metformin exerts other favourable effects^3^. Wulffele *et al*.^4^ comprehensively reviewed the results of 41 randomized-controlled trials in patients with T2DM, and concluded that metformin, independently on its hypoglycemic properties, significantly reduces total and LDL cholesterol. Contrarily, the authors did not find the effects on blood pressure, HDL cholesterol and triglycerides^4^. Metformin is also known to possess anti-inflammatory, anti-oxidative and anti-cancer properties^5,6^.

Metformin has also been found to have beneficial effects on the cardiovascular system. The results of the UKPDS trial showed that metformin can significantly reduce the diabetes-related death (by 42%), and all-cause mortality (by 36%) as well as the risk of myocardial infarction in patients with T2DM (by 39%)^7^. In addition, a UKPDS follow-up study indicated that the cardiovascular risk continued to fall after the study^8^. Comprehensive papers from Hong^9^, Yang^10^, Kinsara^11^, Chi^12^ and Nesti^13^ highlight that metformin attenuates ischemia-reperfusion injury in myocardium and prevents remodelling induced by humoral and hemodynamic factors. Recent publications have also found metformin to demonstrate anti-atherosclerotic^14^ and pro-fibrinolytic properties^1^. For instance, 12-week administration of metformin at a dose of 2550 mg per day contributed to a reduction of PAI-1 levels (200.7 ng/mL at baseline versus 173.7 ng/mL after 12 weeks)^15^. Despite all these beneficial pharmacological activities metformin is characterized by unfavourable pharmacokinetic properties. Due to the slow and incomplete absorption from the gastrointestinal tract, resulting in relatively low bioavailability and considerable inter- and intra-individual differences in clinical response to metformin, several derivatives of this drug have been synthesized to improve the physico-chemical properties of the parent drug. Our recent *in vitro* studies^16–18^ of metformin and its sulfenamide and sulfonamide derivatives have shown that some of the tested biguanides are characterized by beneficial properties with regard to plasma haemostasis, which is frequently impaired in T2DM patients.

Metformin affects positively the functions of the endothelium, mainly by protection from oxidative stress and inflammation, and the negative effects of angiotensin II^19^. Furthermore, metformin appears to attenuate cardiac remodelling by reducing smooth muscle cell proliferation, hypertrophy, and inflammation-induced damage^13,20^. Also other authors confirm beneficial effects of metformin on endothelial function^21–24^. However, as Nesti^13^ highlights the beneficial effects of metformin on endothelial function, proved in an animal model, still have to be convincingly confirmed in humans. The results of one clinical trial have shown treatment with metformin to be associated with improvement in some markers of endothelial functions, including von Willebrand factor (vWF) and vascular cell adhesion molecule 1 (VCAM-1)^21^.

**Figure 1:**
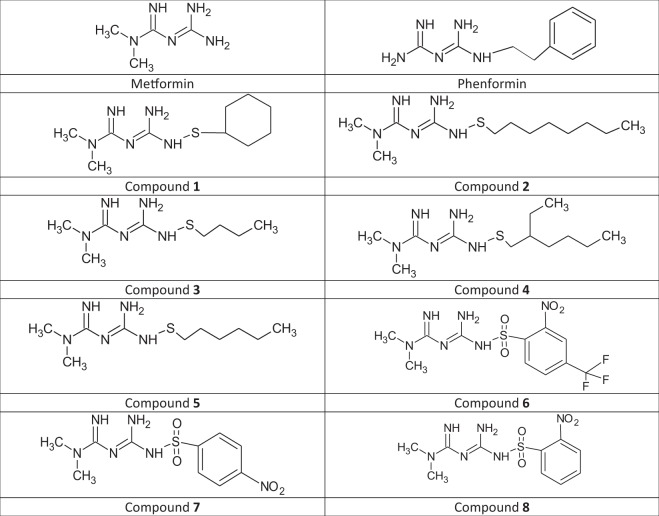
Chemical structure of tested biguanide derivatives – metformin, phenformin and compounds **1–8**.

With the multidirectional effects of metformin on plasma, platelets and vascular haemostasis in mind, the objective of this paper was to assess the effects of metformin, phenformin and eight recently published sulfenamide and sulfonamide derivatives of metformin (Fig. 1) on the selected parameters of vascular and plasma haemostasis. In the first stage of the research, the viability and barrier properties of human umbilical vein endothelial cells (HUVECs) were evaluated. To further characterize the mode of action of biguanides, their effect on apoptosis was determined. Following this, the study examines the effects of biguanides on intracellular levels of tissue factor (TF), release of vWF and tissue plasminogen activator (t-PA) from HUVECs and surface expression of intercellular adhesion molecule 1 (ICAM-1). The final part of the current paper estimates the influence of metformin derivatives on the platelet thrombus formation, and the blood coagulation.

## Results

### The effect of biguanides on the integrity of human endothelium and smooth muscle cells analysed in the RTCA-DP system

**Figure 2:**
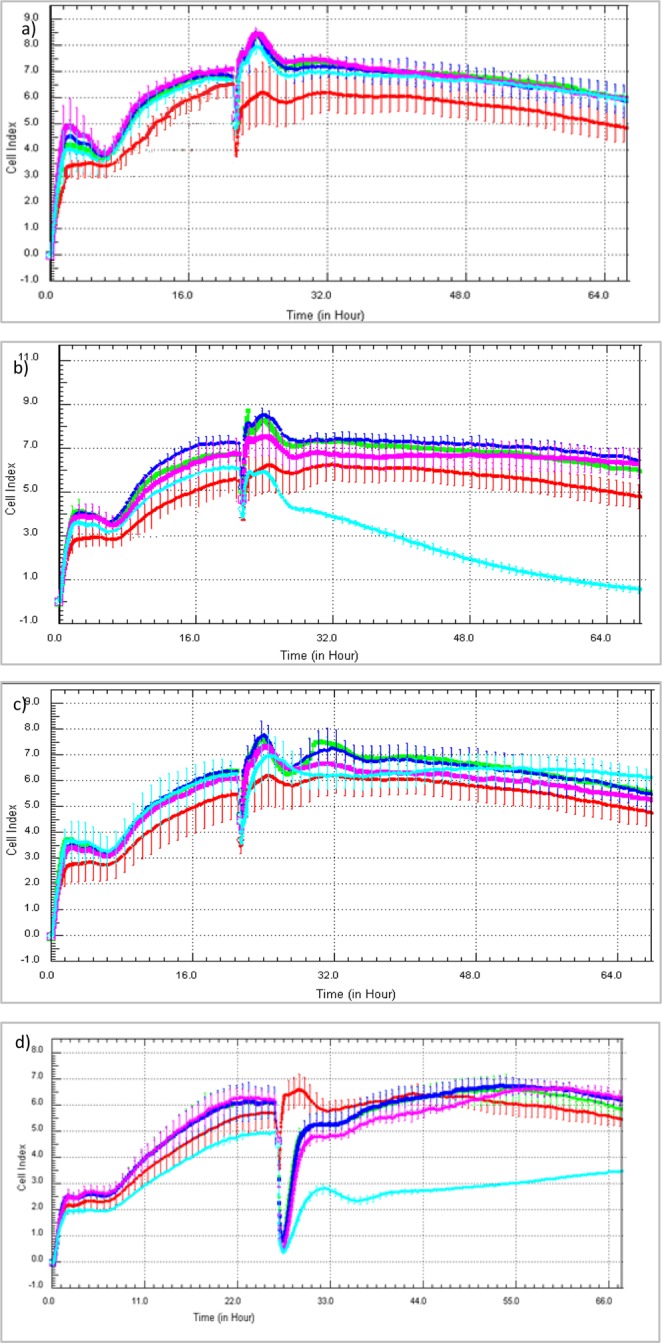
The effect of the exposure of biguanides (**a** – metformin, **b** – phenformin, **c** – comp. 3, **d** – comp. 7) on human vascular endothelial cells (HUVECs) measured by RTCA-DP system. The pictures present representative plots of one experiment conducted in duplicates (the results are presented as a mean (solid line) ± standard deviation). For the statistical analysis there were conducted three independent experiments. Red line – control (unstimulated cells); green line – compounds at the concentration of 0.006 µmol/mL; navy blue line − 0.06 µmol/mL; pink line − 0.3 µmol/mL; light blue line − 1.5 µmol/mL. CI – Cell index.

**Figure 3:**
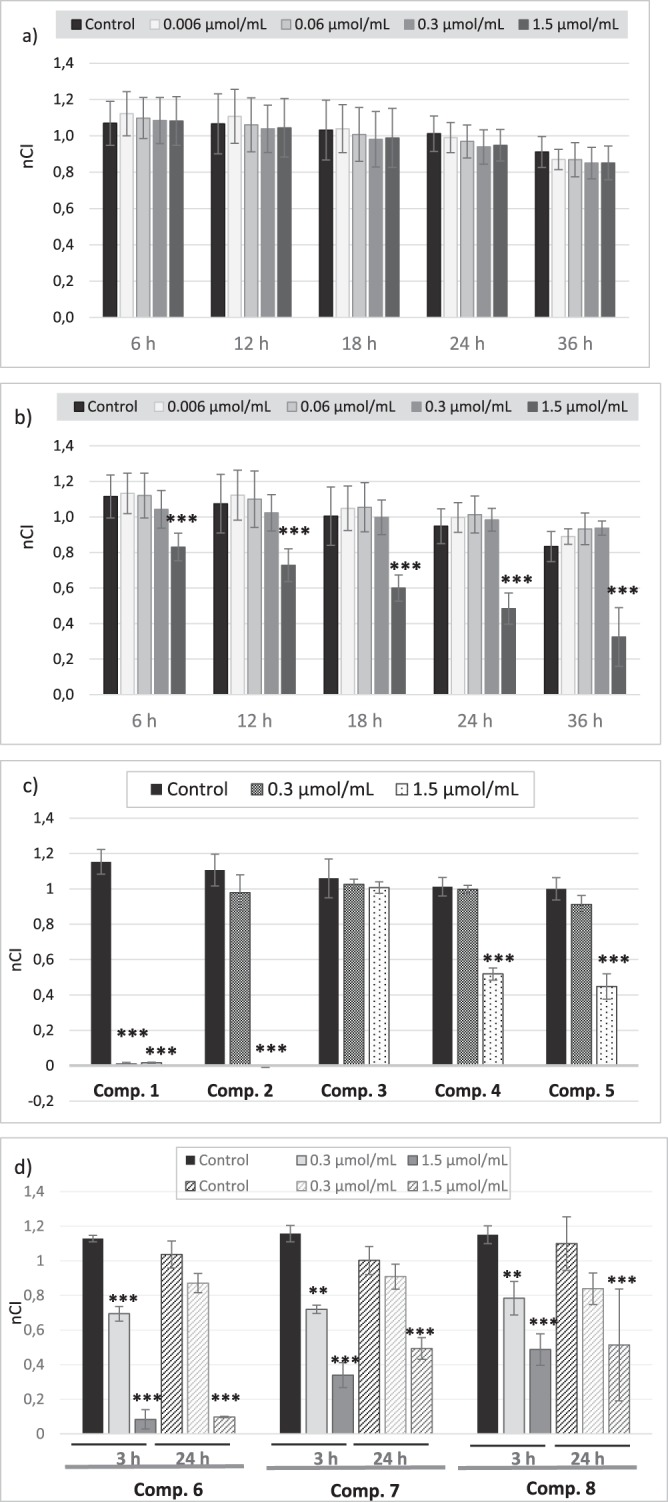
The effect of the stimulation of endothelial cells with biguanides (**a** – metformin, **b** – phenformin, **c** – sulfenamides (1–5), **d** – sulfonamides (6–8)) measured by RTCA-DP at different time-points. The figures present the calculated normalized Cell Index values (nCI).

Upon the stimulation with metformin over the entire concentration range (Figs 2a, 3a, Supplementary Table S1) the normalized cell index (nCI) of the HUVECs increased with regard to that of untreated cells up to 12 hours. However, the reported differences were not of statistical significance (p > 0.05). In the case of phenformin the highest concentration contributed to the significant decrease (p = 0.037) in nCI three hours after the drug addition (Fig. 3b). Despite the fact that phenformin is no longer clinically used, we decided to examine it to see how the presence of the aromatic ring and the lack of N-methyl groups affect the parameters determined. Sulfenamide **1** with cyclohexyl substituent appeared to be the most toxic of all tested compounds since even the lowest concentration (0.006 µmol/mL) was associated with a significant decrease (depending on the time point p = 0.025–0.001) in nCI value (Fig. 3c). In contrast, compound **3** with an *n*-butyl chain, did not affect endothelial integrity at any of the analyzed time-points over the entire concentration range (p > 0.05) (Fig. 3c). Other sulfenamides (compounds **2**, **4** and **5**) significantly decreased endothelial cells (EC) integrity only at the highest concentration tested (1.5 µmol/mL) (p < 0.001). In the case of the sulfonamides, the nCI values fell considerably during three hours, and then returned to control levels. Only the highest concentration sulfonamides demonstrated a significant reduction in nCI values at all time-points of the analysis (p < 0.001) (Fig. 3d).

**Figure 4:**
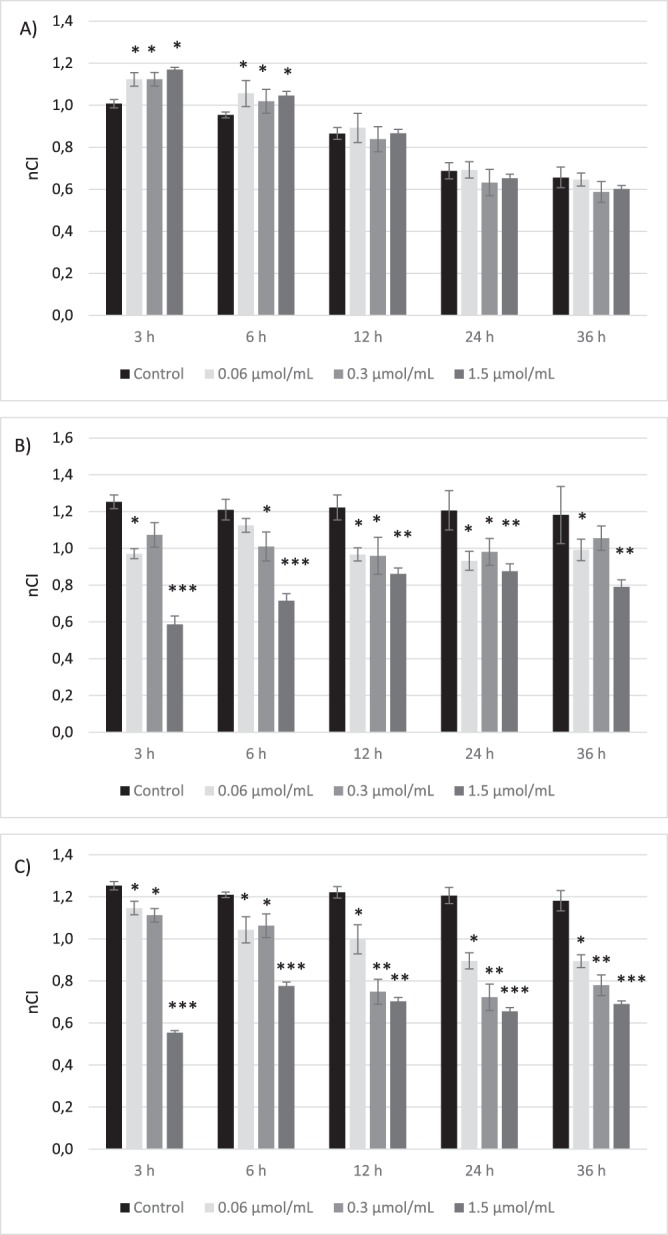
The effect of the stimulation of human aortal smooth muscle cells (AoSMC) with biguanides (**A** – metformin, **B** – sulfenamide 3, **C** – sulfonamide 8) measured by RTCA-DP at different time-points. The figures present the calculated normalized Cell Index values (nCI). The results are presented as mean ± SD, n = 4–5; *p < 0.05, **p < 0.01, ***p < 0.001.

Another part of the experiments was the evaluation of biguanide effects on viability and integrity of human aortal smooth muscle cells (AoSMC). Upon the 6 hours stimulation with metformin over the entire concentration range (Fig. 4) a significant increase in the nCI value was reported. For instance, nCI of the cells treated with metformin increased in comparison with unstimulated cells (control) up to 110% (0.954 vs. 1.046 for 1.5 µmol/mL; p < 0.05). In the case of longer metformin stimulation, no significant changes in nCI values were reported. Sulfenamide **3** with *n*-butyl substituent at the concentration of 1.5 µmol/mL appeared to be toxic towards AoSMC since it has contributed to the significant (p < 0.001) decrease in nCI (Fig. 4B). The results of sulfonamides (compounds **6**–**8**) represent mostly similar tendency. The stimulation of AoSMC with the highest tested concentration (1.5 µmol/mL) contributed to the significant decrease in nCI value during the entire incubation time (36 hours). For instance, sulfonamide **8** at concentration of 1.5 µmol/mL decreased nCI of control sample by 42% (0.689 ± 0.07 vs. 1.181 ± 0.156 for control) (Fig. 4C).

### HUVEC and AoSMC morphology

**Figure 5:**
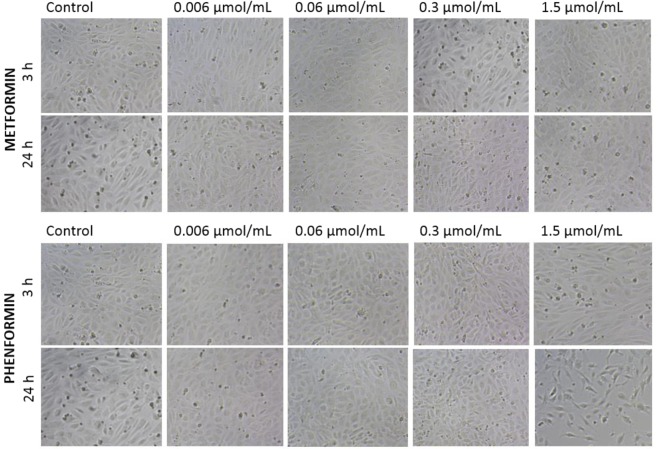
Time- and dose-dependent effect of metformin and phenformin on endothelial cell (HUVECs) viability and integrity. HUVECs were cultured in the presence of metformin or phenformin at concentrations of 0.006–1.5 μmol/mL; cultures in medium alone were also used as controls. Representative phase-contrast cell images are shown after three and 24 hours of incubation (100-fold magnification).

The effects of biguanides on EC viability and integrity were also monitored using light and phase-contrast microscopy. Figure 5 depicts the dose-dependent effects of metformin and phenformin on ECs growth after three and 24 hours of incubation. Metformin did not affect cell viability, shape and integrity over the entire concentration range while 1.5 µmol/mL phenformin decreased viable cells numbers and density.

**Figure 6:**
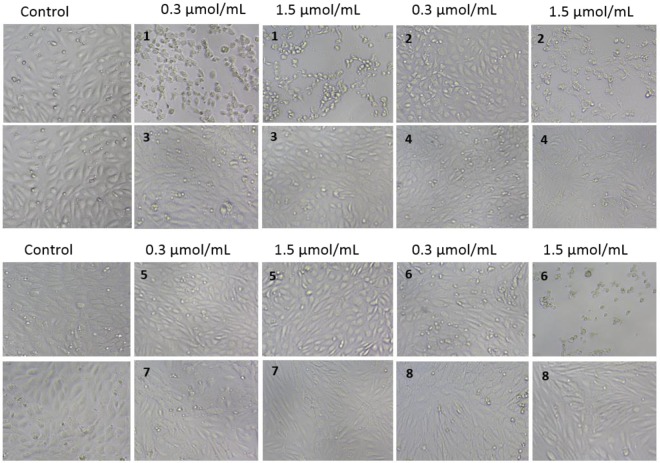
Dose-dependent effect of biguanides (compounds 1–8) on endothelial cell (HUVECs) viability and integrity after 24-hour incubation. HUVECs were cultured without (control) and in the presence of compounds 1–8 at concentration of 0.006–1.5 μmol/mL. Representative cell images are shown for concentration 0.3 and 1.5 μmol/mL (100-fold magnification).

Images in Fig. 6 present the effects of sulfenamides and sulfonamides on HUVEC growth and morphology after 24 hours of treatment. In the case of compound **1**, morphological examination of HUVECs showed severe compound-mediated changes manifested by membrane disruption, cell shrinkage and cytoplasm leakage at concentrations of 0.3 and 1.5 µmol/mL. A similar effect, although to a lesser extent, was also observed for the highest concentration of compound **2**. Sulfenamide with *n*-butyl chain (compound **3**) did not exert any significant effect on the viability, integrity and shape of the cells over the entire concentration range. Similarly, incubation of the cells with compound **4** or **5** did not reveal substantial morphological changes but a tendency for cell lengthening could be observed in the case of the highest concentration (1.5 µmol/mL) of compound **4**. Compound **6** exhibited an unfavourable effect on the cells viability and integrity at 1.5 µmol/mL, demonstrated by cell shrinkage and clumping.

The cells treated with sulfonamides **7** and **8** did not exhibit any profound morphological changes; however, at the highest concentration, more elongated cells were recognized in comparison with controls.

**Figure 7:**
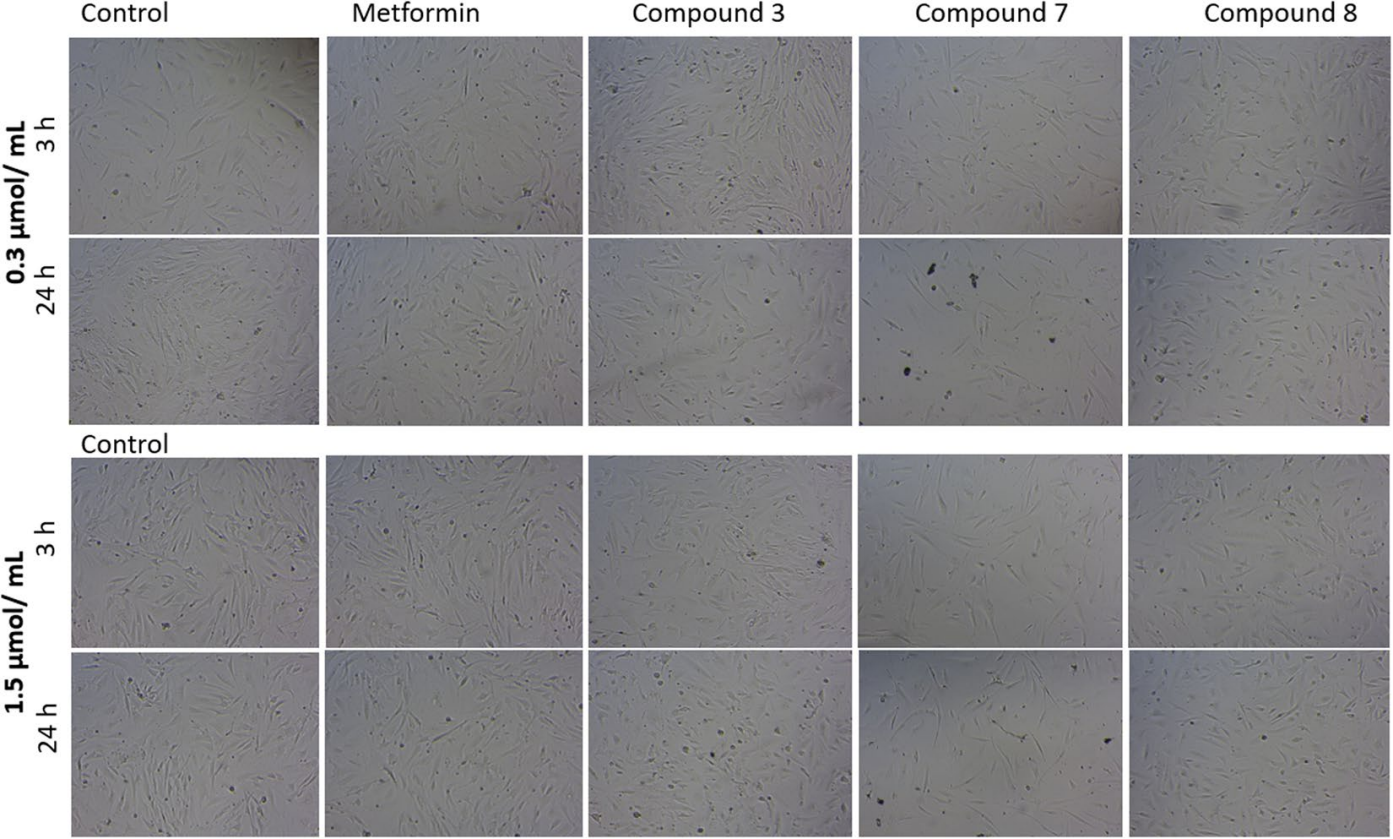
Dose-dependent effect of selected biguanides on aortal smooth muscle cells (AoSMC) viability and integrity after 3- and 24-hour incubation. AoSMCs were cultured without (control) and in the presence of biguanides at concentration of 0.006–1.5 μmol/mL. Representative cell images are shown for concentration 0.3 and 1.5 μmol/mL (100-fold magnification).

The effects of biguanides on morphology of AoSMC were also monitored using both light and phase-contrast microscopy (Fig. 7). Metformin, similarly to HUVECs, did not affect AoSMC viability, shape and integrity over the entire concentration range. Incubation of AoSMC with 1.5 µmol/mL of compound **3** resulted in substantial decrease in the number of attached cells and increase numer of shrinked and rounded cells. In the case of compound **7** a tendency towards cells lengthening was reported at the concentration range 0.3–1.5 µmol/mL after 24 hours incubation. In turn, stimulation of AoSMC with sulfonamide **8** over the entire concentration range did not contribute to the significant changes in cellular morphology.

### Apoptosis

**Figure 8:**
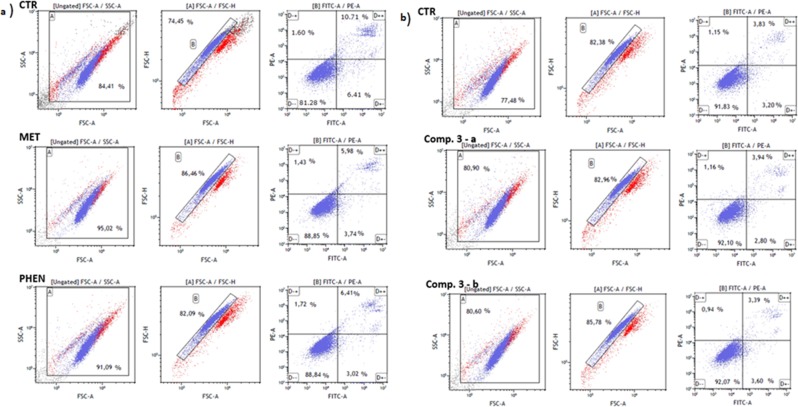
The effect of selected biguanides on HUVECs death. (**a**) Representative histograms of unstimulated HUVECs (control, CTR), metformin (0.3 μmol/mL) and (phenformin 0.3 μmol/mL) displaying the percentage of cells gathered within established gates. (**b**) Representative histograms of unstimulated HUVECs (control, CTR), and compound 3 at the concentration of 0.06 μmol/mL (comp. 3 – **a**) and 0.3 μmol/mL (comp. 3 – **b**) displaying the percentage of cells gathered within established gates. FSC-A vs SSC-A plots were used for gating cells and to identify any changes in the scatter properties of the cells. Annexin V FITC-A (x-axis) vs Propidium Iodide (y-axis) plots from the gated cells show the populations corresponding to living cells (Annexin V(−) and PI (−)) (D–); early apoptotic cells (Annexin V (+) and PI (−)) (D+−), late-apoptotic cells (Annexin V(+) and PI (+)), and necrotic cells (Annexin V (−) and PI (+)) (D−+).

Metformin, phenformin and compound **4** were found to increase the total number of cells gathered within gates A and B, therefore giving evidence on decreased apoptosis of HUVEC cells (p < 0.05) (Table 1, Fig. 8). Furthermore, stimulation of HUVECs with metformin and phenformin was associated with a higher percentage of viable cells (↑ AV− PI−) (p = 0.008 and p = 0.015, respectively). On the contrary, compound **2** at the concentration of 1.0 µmol/mL contributed to a decrease in the percentage of cells within gates A and B (p = 0.001 and p < 0.001, respectively). In addition, the percentage of viable cells (AV− PI−) was reduced in comparison with control (30.8 ± 2.3% vs. 90.2 ± 2.4%; p < 0.001), and the population of necrotic cells (AV− PI+) increased approximately 10-fold (12.6 ± 3.8% vs. 1.4 ± 0.3%; p = 0.040).

**Table 1:** Annexin V-FITC/PI double staining analysis of apoptosis in HUVECs.

Compound	Conc. [µmol/mL]	Gate A [%]^a^	Gate B [%]^b^	D− [%]^c^	D− + [%]^c^	D + − [%]^c^	D + + [%]^c^
**Control I**	—	83.6 ± 0.9	73.1 ± 1.2	80.0 ± 1.1	1.6 ± 0.2	8.1 ± 1.6	10.4 ± 0.6
**Metformin**	0.06	87.1 ± 3.4	**78**.**3** ± **3**.4*	83.4 ± 2.5	1.8 ± 0.2	**4**.**0** ± **0**.6*	10.9 ± 1.9
	0.3	**93**.**2** ± **1**.8**	**83**.**6** ± **1**.8*	**88**.**0** ± **1**.5**	1.5 ± 0.0	**4**.**0** ± **0**.3*	**6**.**5** ± **1**.3*
**Phenformin**	0.3	**89**.**6** ± **1**.7*	**79**.**4** ± **2**.4*	85.4 ± 4.1	1.8 ± 0.1	**4**.**6 ± 2**.1*	8.2 ± 2.1
	1.0	**90**.**8** ± **0**.3**	**86**.**4** ± **2**.0**	**85**.**7** ± **1**.6*	1.4 ± 0.1	**5**.**0** ± **0**.6*	7.8 ± 1.2
**Control II**	—	75.6 ± 1.9	82.4 ± 0.3	90.2 ± 2.4	1.4 ± 0.3	3.6 ± 0.8	4.8 ± 1.3
**Comp. 1**	0.006	81.0 ± 3.3	87.4 ± 2.5	92.0 ± 0.7	1.0 ± 0.1	**2**.**8** ± **0**.3*	4.2 ± 0.3
**Comp. 2**	0.3	87.3 ± 3.6	**94**.**9** ± **0**.5***	93.8 ± 0.7	1.2 ± 0.3	2.3 ± 0.1	2.7 ± 0.5
	1.0	**37**.**8** ± **2**.7**	**41**.**6** ± **2**.1**	**30**.**8** ± **2**.3***	**12**.**6** ± **3**.8*	**14**.**2** ± **3**.7*	**42**.**5** ± **6**.8**
**Comp. 3**	0.06	79.2 ± 3.3	82.5 ± 0.6	92.3 ± 0.2	1.2 ± 0.3	2.4 ± 0.4	4.2 ± 0.2
	0.3	78.5 ± 2.1	**85**.**3** ± **2**.3**	90.9 ± 1.6	1.2 ± 0.2	3.9 ± 0.6	4.1 ± 0.9
**Comp. 4**	0.3	**85**.**2** ± **2**.3*	**93**.**9** ± **0**.5**	91.9 ± 1.9	1.2 ± 0.4	4.1 ± 1.1	2.8 ± 0.5
	1.0	**83**.**6** ± **1**.2*	**86**.**8** ± **0**.8*	**80**.**1** ± **1**.3*	1.5 ± 0.1	6.9 ± 0.8	**11**.**5** ± **0**.8*
**Control III**	—	85.0 ± 0.9	80.5 ± 2.3	88.1 ± 3.8	1.5 ± 0.3	5.1 ± 1.6	5.2 ± 2.0
**Comp. 5**	0.3	88.6 ± 1.9	**88**.**9** ± **2**.6*	90.3 ± 2.8	1.2 ± 0.1	4.1 ± 0.6	4.4 ± 0.4
	1.0	87.5 ± 2.6	**89**.**0** ± **1**.1*	89.5 ± 1.4	1.3 ± 0.2	4.4 ± 0.8	4.9 ± 0.7
**Comp. 6**	0.06	84.2 ± 0.6	82.5 ± 1.9	90.1 ± 1.2	1.3 ± 0.2	4.3 ± 1.1	4.3 ± 0.3
	0.3	84.7 ± 2.2	82.6 ± 0.9	90.2 ± 1.4	0.8 ± 0.2	4.5 ± 1.0	4.5 ± 0.4
**Comp. 7**	0.3	85.6 ± 3.8	**86**.**5** ± **4**.3*	89.0 ± 0.5	1.7 ± 0.7	5.0 ± 0.9	4.3 ± 0.5
	1.0	85.2 ± 6.2	**83**.**7** ± **1**.7*	86.7 ± 2.4	1.4 ± 0.6	4.7 ± 0.6	7.1 ± 1.9
**Comp. 8**	0.3	86.1 ± 1.9	**83**.**4** ± **2**.4**	85.3 ± 2.0	**7**.**9** ± **0**.6***	3.1 ± 1.1	3.6 ± 0.5
	1.0	87.2 ± 1.9	80.3 ± 1.8	**72**.**0** ± **0**.5*	**19**.**6** ± **1**.0**	3.0 ± 0.3	5.4 ± 0.5

Endothelial cells were treated with various concentrations of biguanides for 24 hours followed by staining with Annexin V FITC and propidium iodide (PI). The concentration for apoptosis were chosen on the basis of results collected in viability and integrity test. ^a^Cells gathered within the gate A reflecting the % of the absolute number of acquired events; ^b^the percentage of the cells collected in gate A which are singlets (detected using FSC-A/FSC-H plots); ^c^the cells gathered in gate B were divided depending on the staining with Annexin V and PI: (D–) − living cells, (D −+) – necrotic cells; (D +−) – early-apoptotic cells; (D ++) – late-apoptotic cells. The results are presented as mean ± standard deviation (SD), n = 4. The values given in bold represent statistically significant (*p < 0.05; **p < 0.01; ***p < 0.001) changes versus respective controls (metformin, phenformin – control I; compounds **1**–**4** – control II, compounds **5**–**8** – control III).

Compounds **3** and **5** did not affect HUVEC apoptosis and necrosis, as the percentages of Annexin V+ (AV+) Propidium iodide - (PI−) cells or apoptotic (AV + PI+) cells and necrotic (AV− PI+) cells, were comparable to those of unstimulated HUVECs (p > 0.05). Simultaneously, compounds **3** and **5** were not observed to have any effect on cell viability since the percentage of AV− PI− did not differ from controls. Similar effects were found for compound **6**. Sulfonamide **7** did not affect the percentage of viable, apoptotic and necrotic cells, while its isomer (sulfonamide **8**), at a concentration of 1.0 µmol/mL, led to a significant decrease in viable cells (72.0 ± 0.5% vs. 88.1 ± 3.8%, p = 0.019) and an increase in the percentage of necrotic cells (19.6 ± 1.0% vs. 1.5 ± 0.3%; p = 0.001).

### Tissue Factor

**Figure 9:**
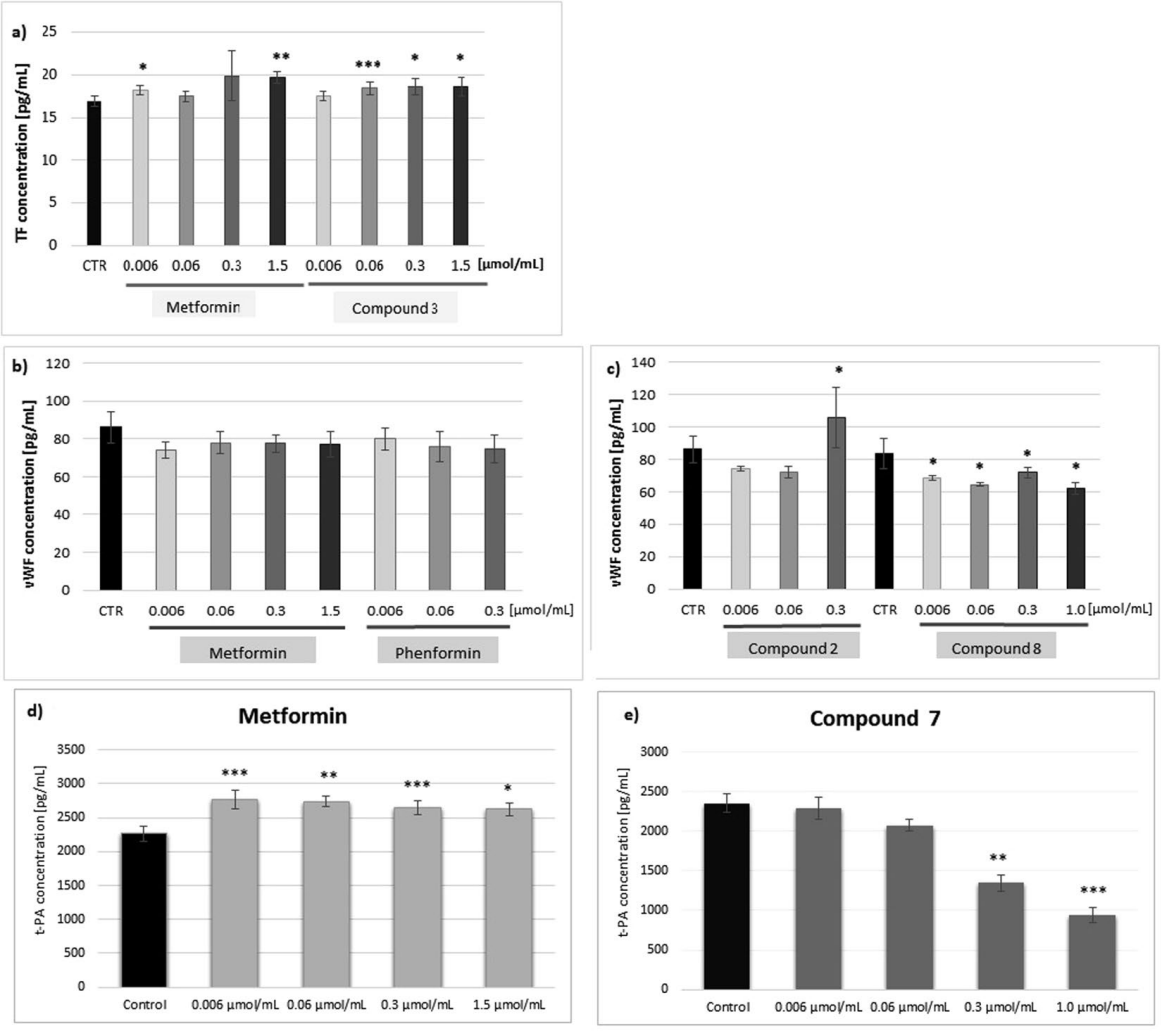
The effects of metformin and biguanide derivatives on selected markers of endothelial function. (**a)** The effects of metformin and *n*-butyl sulfenamide (comp. 3) on the total cellular production of TF in undisturbed HUVEC cells. The results are presented as mean ± SD; n = 4; *p < 0.05; **p < 0.01; ***p < 0.001. The quantification of TF in cell lysates was conducted using immunohistochemichal ELISA test. Metformin over the entire concentration range contributed to the increase in the intracellular TF level, however, the significant results were reported for 0.006 and 1.5 μmol/mL. Compound 3 also elevated the concentration of intracellular TF. (**b**,**c)** The effects of metformin, phenformin (**a**) and n-octyl sulfenamide (comp. 2) and sulfonamide 8 (comp. 8) (**b**) on the release of von Willebrand Factor from HUVEC cells. The results are presented as mean ± SD; n = 4–6, * denotes p < 0.05. The quantification of vWF in cell culture supernatants was carried out using immunohistochemichal ELISA test. Metformin and phenformin over the entire concentration range contributed to the decrease in the amount of released vWF, however, the changes were not statistically significant (p > 0.05). Compound 2 was shown to exert complex effects on vWF release, while compound 8 decreased the concentration of vWF in cells supernatants over the entire concentration tested. (**d**,**e)**. The effects of metformin (**d**), and sulfonamide 7 (**e**) on the release of t-PA from undisturbed HUVEC cells. The results are presented as mean ± SD; n = 4, ** denotes p < 0.01; ***p < 0.001. The quantification of t-PA in cell culture supernatants was carried out using immunohistochemical ELISA test. Metformin over the entire concentration range contributed to the significant increase in the amount of released t-PA (p < 0.05). Compound 7 was shown to decrease t-PA release at the concentration of 0.3 and 1.0 µmol/mL.

It was found that among the tested compounds metformin, sulfenamide **3** (Fig. 9a) and sulfenamide **4** (Supplementary Fig. S1) contributed to the increase in TF production in HUVEC cells (p-value varying from 0.033 to 0.001). In the case of compound **5**, an increase in TF production was reported (18.31 ± 0.64 pg/mL for 0.3 µmol/mL vs. 16.97 ± 0.60 pg/mL for control); however, the changes were not statistically significant (p = 0.052). Neither phenformin, sulfonamide **7** or sulfonamide **8** affected the intracellular level of TF. Compound **1**, tested only at a dose of 0.006 µmol/mL because of its unfavourable effect on HUVECs, did not change the TF concentration. Similarly, compound **6** was found not to influence intracellular TF level within the concentration range 0.006–0.06 µmol/mL (p > 0.05).

### Von Willebrand Factor (vWF)

Metformin and phenformin were found to decrease the release of vWF from undisturbed ECs over the entire concentration range; however, the differences were not of statistical significance (p > 0.05) (Fig. 9b,c). Compounds **4**, **6** and **7** did not influence the concentration of released vWF, while compounds **3** and **5** contributed to its significant increase at the highest concentration tested (p = 0.025 and p = 0.015, respectively) (Supplementary Table S3). Compound **8** significantly reduced the amount of released vWF by approximately 20% in comparison to control (p = 0.027). While derivative **2** with an *n*-octyl chain decreased the amount of released vWF at lower concentrations (p = 0.068), it was found to significantly elevate vWF level at 0.3 µmol/mL (105.65 ± 18.35 pg/mL vs. 86.08 ± 8.23 pg/mL for control, p = 0.05).

### Tissue activator plasminogen (t-PA) release

While metformin was associated with increased release of t-PA from undisturbed ECs across the entire concentration range (Fig. 9d) (p-value range 0.022–0.001, depending on the metformin concentration), phenformin was not found to have any significant effect on t-PA concentration in cell supernatants (Supplementary Table S4). The other tested compounds exhibited the same pattern of influence regarding t-PA release; neither sulfenamides nor sulfonamides influenced the amount of t-PA in HUVEC supernatants at lower concentrations; however, they significantly reduced the level at higher concentrations. For instance, derivative **7** at 0.3 and 1.0 µmol/mL significantly decreased t-PA concentration (947.1 ± 124.8 pg/mL at 1.0 µmol/mL vs. 2356.5 ± 70.5 pg/mL for control, p < 0.001, respectively) (Fig. 9e).

### Effects on ICAM-1 expression

**Figure 10:**
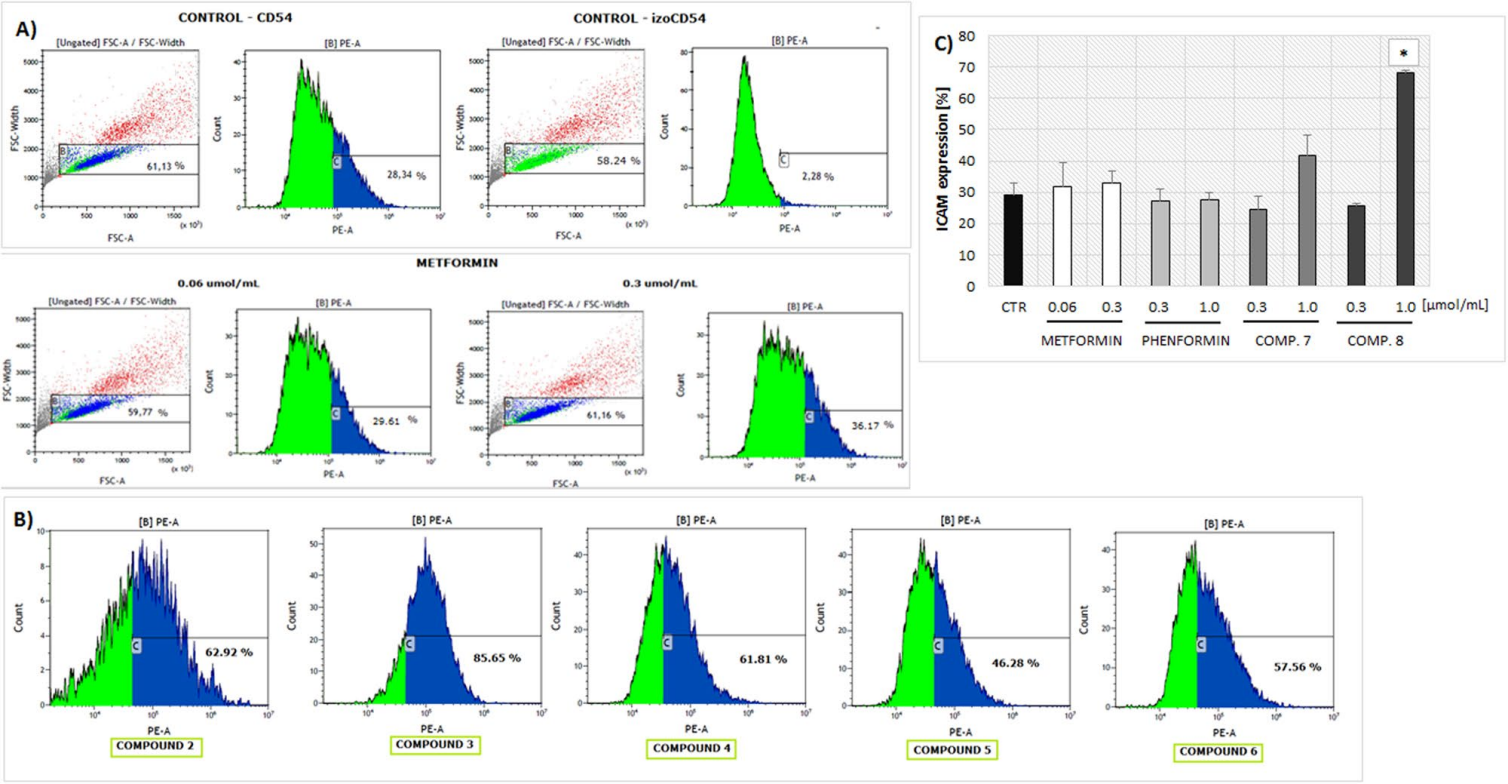
The effect of biguanide derivatives on ICAM-1 expression on HUVECs. (**A**) Representative histograms of unstimulated HUVECs (control – CD54, and isotype control - izoCD), and cells treated with metformin (0.06 and 0.3 μmol/mL) displaying the percentage of cells in gate B (left pictures) and percentage of cells expressing ICAM (right image). (**B**) The effects of compounds 2–6 at the highest concentrations on the percentage change of the surface ICAM-1 expression (representative histograms). (**C**) The summary of effects of metformin, phenformin, compounds 7 and 8 on the ICAM-1 expression (n = 3–6), mean ± SD; ***p < 0.05.

As presented in Fig. 10a,c and Supplementary Table S5 neither tested metformin concentration significantly affected the expression of ICAM-1 on the surface of HUVECs in comparison to unstimulated cells (p > 0.05). Similarly, the second tested biguanide, previously available phenformin was not found to influence ICAM-1 expression (Fig. 10c). All tested compounds apart from sulfonamide **7** (0.3 and 1.0 µmol/mL) and **8** (0.3 µmol/mL) significantly increased the expression of ICAM-1 on the EC surface (Fig. 10b).

### Thrombus formation

**Figure 11:**
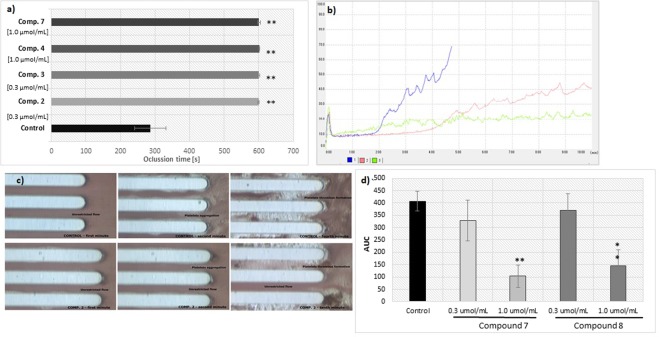
Evaluations of effects of selected biguanides on the platelets-dependent thrombus formation. A collagen coated microchip (Platelet Chip; PL-Chip) was used for analysis of platelet-dependent thrombus formation at 2000 s^−1^. (**A**) The effects of sulfenamides 2, 3, 4 and sulfonamide 7 on the occlusion time (OT). The results are presented as mean ± SD; n = 3, ** denotes p < 0.01. (**B**) Representative plots for T-TAS data obtained in healthy control sample (blue colour) and after stimulation with compound 2 (0.3 μmol/mL) (light pink) or compound 3 (0.3 μmol/mL) (green). X-axis – time (minutes); Y-axis – pressure (kPa). (**C**) Camera image of blood flow and platelet thrombus formation in control sample and sample treated with compound 2 (0.3 μmol/mL). (**D**) Concentration-dependent effects of compounds 7 and 8 on the area under the curve (AUC). The results are presented as mean ± SD; n = 3, ** denotes p < 0.01.

Metformin at both tested concentrations did not affect the onset of platelet thrombus formation (T_10_ constant, p > 0.05), but at 0.3 µmol/mL produced prolongations of OT by 1.21-fold (p = 0.031) and CT by 1.37-fold (p = 0.025) (Supplementary Table S6). Metformin contributed to a decrease in AUC_10,_ however the changes were not statistically significant (p > 0.05). Most of the tested biguanides appear to prevent platelet-dependent thrombus formation which were demonstrated by prolonged T_10_, OT and CT parameters as well as decreased AUC_10_. In particular, derivatives **4**, **7** and **8** contributed to the significant prolongation of T_10_. All examined biguanides, depending on the concentration, significantly prolonged OT, however, it should be noted that compounds **2**, **3**, **4**, and **7** contributed to incomplete occlusion of capillaries, as indicated by an OT exceeding 600 seconds (the maximal length of analysis) (p < 0.01) (Fig. 11a–c). The anti-coagulation properties of sulfenamides and sulfonamides was also revealed by a decreased AUC_10_ value. For instance, compounds **7** and **8** at 1.0 µmol/mL decreased AUC_10_ by 3.93- (p = 0.018) and 2.82-fold (p = 0.025), respectively (Fig. 11d).

**Figure 12:**
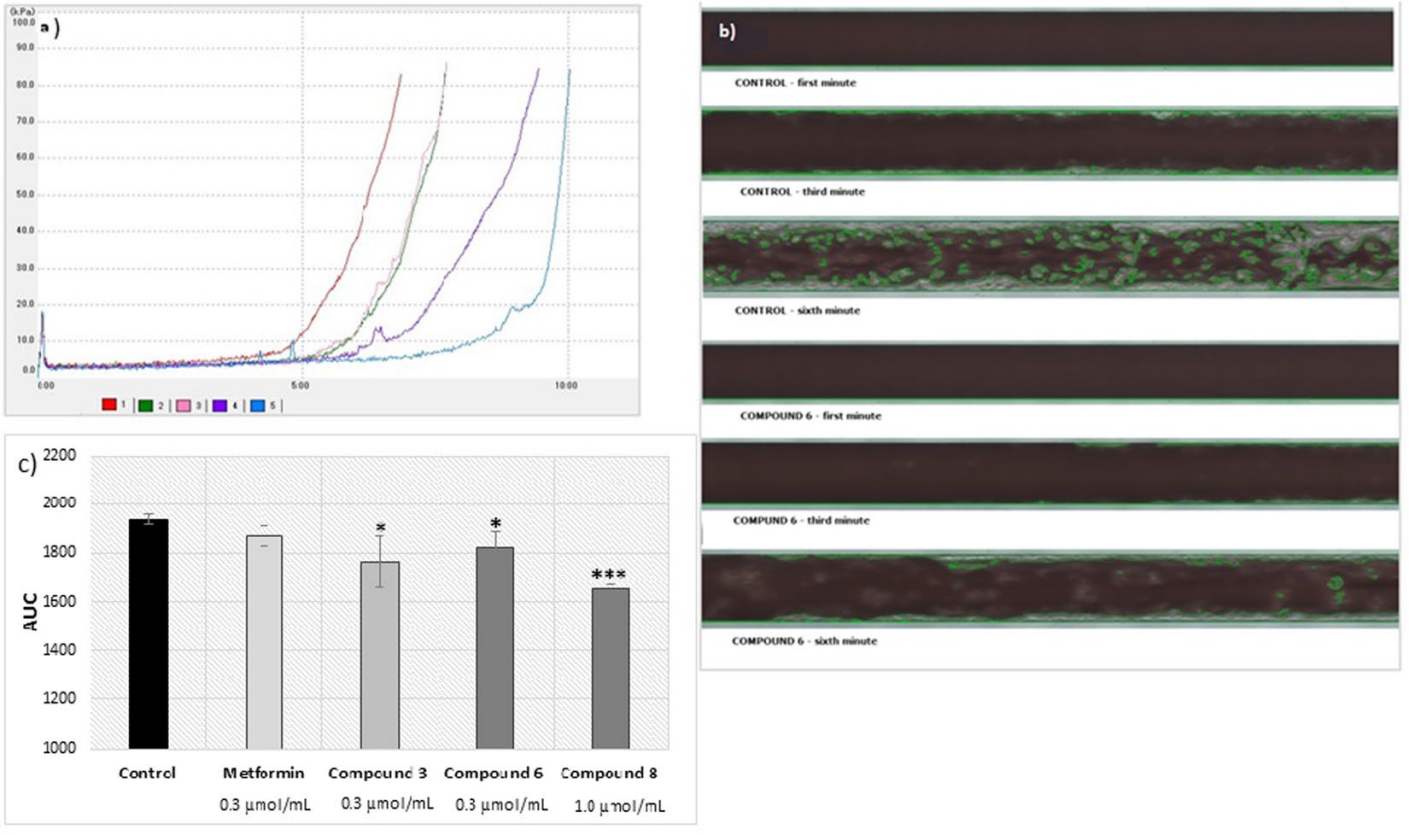
Evaluations of effects of selected biguanides on the white thrombus formation. A collagen and thromboplastin coated microchip (Atheroma Chip; AR-Chip) was used for analysis of fibrin rich platelets thrombus formation at 600 s^−1^. (**a**) Representative plots for T-TAS data obtained in healthy control sample (red colour) and after stimulation with metformin (0.3 μmol/mL) (green), compound 3 (0.3 μmol/mL) (pink), compound 6 (0.3 μmol/mL) (purple) or compound 8 (1.0 μmol/mL) (blue colour); X-axis – time (minutes); Y-axis – pressure (kPa). (**b**) Camera image of blood flow and white thrombus formation in control sample and sample treated with compound 6 (0.3 μmol/mL). The formation of white thrombus is marked with green lines. (**c**) Concentration-dependent effects of metformin and compounds 3, 6 and 8 on the area under the curve (AUC). The results are presented as mean ± SD; n = 3, * denotes p < 0.05; ***p < 0.001.

Studies using collagen and thromboplastin coated AR chips revealed that metformin (Supplementary Fig. S3, Supplementary Table S7) does not significantly influence the process of fibrin-rich platelet thrombus formation, as indicated by constant values for parameters T_10_, OT_80_, T_10–80_ and AUC_30_ (p > 0.05). More prominent treatment effects were seen for the other tested biguanide: in particular, compound **6** prevented the onset of white thrombus formation (360.7 ± 25.8 s for 0.3 µmol/mL versus 301.7 ± 21.2 s for control; p = 0.023) (Fig. 12a,b). Sulfenamide **3** and sulfonamides **6** and **8** also demonstrate anti-coagulation properties, as indicated by their reducted AUC_30_ values (Fig. 12c).

A summary table was prepared to facilitate the analysis of collected results (Table S8. Supplementary materials).

## Discussion

### Viability, integrity and morphology of human endothelium and aortal smooth muscle cells

Type 2 diabetes mellitus (T2DM) is a multi-factorial disease and can cause multiple organ dysfunction through several mechanisms^23^, including systemic insulin resistance, which promotes hyperglycemia and dyslipidemia^25^. It has been reported that these metabolic abnormalities account for increased risk of vascular disease which is a major cause of morbidity and mortality among patients suffering from T2DM^25^. Therefore, treatment with a drug decreasing insulin resistance, such as metformin, may improve several of primary pathophysiological abnormalities, including insulin resistance, and endothelial function^18^. Apart from its glucose-lowering properties metformin was also found to markedly improve endothelial-dependent vasodilation, and simultaneously reduce the expression levels of dysfunctional biomarkers, such as ET-1, PAI-1, and CRP in endothelial cells^26^. It has been previously reported that metformin might favourably influence plasma haemostasis by increasing antithrombin III activity^18^, and that some of its recently-synthesized derivatives may also exert beneficial effects on selected parameters of plasma haemostasis^16,17^. Based on previously published findings indicating vascular endothelium dysfunction in T2DM subjects^25^ and the promising results of our own previous trials^16,17^, the aim of this study was to further characterize the mode of action of metformin, phenformin and recently synthesized sulfenamides and sulfonamides on selected parameters of endothelial function. Sulfenamide derivatives of metformin are bio-reversible guanidines (N-S) with prodrug properties^27,28^, while sulfonamide derivatives were designed to remain intact in the bloodstream until they are conveyed into the hepatocytes where they can be bioconverted to metformin^29^.

Considering the above-mentioned examples of beneficial effects of metformin on vascular endothelium, the present study used an *in vitro* model based on the Real-Time Cell Electric Impedance Sensing system (RTCA-DP) to determine the potential influence of metformin on endothelial cell integrity. The applied system allows the status of adherent cells to be evaluated by continuous measurements of their integrity and for the immediate and delayed responses to the stimulant to be observed^30^. Our findings indicate that metformin at the concentration range 0.006–0.3 μmol/mL which include also therapeutic plasma concentrations^31^, depending on the stimulation time caused up to approximately 7% increase of endothelial integrity as compared to unexposed cells (Fig. 3); however these changes were not of statistical significance. Microscopic studies also confirmed that metformin does not affect the morphology of endothelial cells (Fig. 5). To the best of our knowledge, few studies have dealt with the effects of metformin on the viability and integrity of endothelial cells using this type of real-time monitoring system of cell status. For instance, based on an end-point test, Esfahanian *et al*.^32^ report that metformin does not affect the integrity of HUVEC cells and its anti-proliferative effect is not associated with cytotoxicity. In another study, Arunachalam *et al*.^33^ used a cell culture model with mouse microvascular endothelial cells to demonstrate that the protective effect of metformin against hyperglycemia-induced endothelial dysfunction was partly due to its effects on sirtuin-1 (SIRT1) expression and activity. Numerous preclinical and clinical studies have shown that metformin exerts favourable hemodynamic and rheological effects in patients with diabetes and other cardiovascular risk factors^21^. For instance, Majithiya and Balaraman^34^ revealed that metformin administration to rats with streptozocin-induced diabetes lowers blood pressure and restores endothelial function.

Similarly to metformin, compound **3** with *n*-butyl chain did not induce the changes in endothelial integrity at any of the analysed time-points over the entire concentration range (Fig. 3) when compared to unstimulated cells. In contrast, other sulfenamides with an alkyl chain longer than four carbon atoms (compounds **2**, **4** and **5**) significantly decreased EC integrity at concentration of 1.5 µmol/mL, which is approximately 3-fold higher than expected plasma concentration. Hence it appears that more profoundly unfavourable effects on cell integrity manifested by cell shrinkage and membrane disruption were observed for sulfenamides with longer alkyl chain in sulfenamides and more lipophilic properties (Figs 3, 6). In the case of all sulfonamides, nCI values decreased profoundly immediately after administration; however, they returned to control values after several hours of co-treatment.

Even less is known on the effects of metformin and other biguanide on viability of human aortal smooth muscle cells (AoSMC). In our studies using XCelligence system it was found that metformin up to 1.5 µmol/mL contributes to significant increase in cellular integrity and adherence. Available data regarding metformin effects on smooth muscle cells are mainly focused on cells proliferation and migration. For instance, Li *et al*.^35^ found that treatment of smooth muscle cells (HASMCs) with metformin inhibited the effect of leptin on HASMC proliferation and metalloproteinase (MMP-2) expression. Also Hao *et al*.^36^ confirmed that metformin suppresses proliferation and migration of HASMC. Our experiments also showed that metformin inhibits AoSMC migration in wound healing assay (data not shown). However, there is no available data on the real-time effects of metformin on smooth muscle cells integrity. Therefore, this is the first study examining the continuous influence of metformin and other biguanides on the AoSMC functions. Most of biguanide derivatives at 1.5 µmol/mL contributed to the significant decrease in nCI value which reflects cellular integrity. However, morphological examination of AoSMCs did not show substantial changes in cells shape for all tested compounds (Fig. 7). These results are in accordance to those collected in experiments on HUVECs.

Since inhibition of cell proliferation and viability can result in apoptosis, the next step of our studies was to determine whether biguanide derivatives affect EC growth by induction of apoptosis. It was found that stimulation of HUVECs with metformin increases the percentage of viable cells (AV− PI−) and decreases the number of early- and late-apoptotic cells (Table 1). These results are in agreement with those obtained using the RTCA-DP system where a slight increase in nCI was reported for the cells stimulated with metformin. Inhibition of apoptosis in HUVEC cell line under conditions mimicking hypoxia-hyperglycemia was also described by Bakhashab *et al*.^37^. Similarly, metformin at the concentration of 100 µmol/L prevented hyperglycemia-promoted apoptosis in HUVECs^38^. These favourable effects of metformin on ECs may provide an explanation for its long-term protection on diabetes-related vascular complications. Interestingly, contrasting results regarding the effects of metformin on apoptosis have been returned by many *in vitro* studies using cancer cell lines^39,40^. This might be due to the concentration of the drug. For example, Queiroz *et al*.^40^ found that at a dose of 10 mM metformin inhibited the proliferation of breast cancer cells (MCF-7 cell line) by promoting cell cycle arrest in the G_0_-G_1_ phase, inhibiting cyclin D1 and inducing cell apoptosis and necrosis. The authors list also other studies which have reported that metformin can increase apoptosis of breast, colon and endometrial cancer cells^40^. In contrast, some studies indicate that metformin did not induce apoptosis^41^. Compound **2** with *n*-octyl chain induced a significant decrease in the percentage of viable cells (AV− PI−), and an increase in the population of necrotic cells (AV− PI+) (Table 1). Most of the other examined compounds, apart from sulfonamide **8**, did not exert any effects on the percentage of viable, apoptotic or necrotic cells which suggest their biocompatibility towards endothelial cells.

### Endothelial cells function, TF production, vWF and t-PA release, and ICAM-1 expression

Tissue factor (TF) is a crucial factor initiating blood coagulation and the extracellular domain of TF serves as a cell surface receptor for coagulation factors VII and VIIa^42^. The current study presents the effects of metformin and its sulfenamide and sulfonamide derivatives on the intracellular production of TF. It was found that metformin and compounds **3** and **4**, which exerted a favourable effect on HUVEC cells demonstrated by RTCA-DP and apoptosis assay, contributed to the elevated TF production in HUVEC cells (Fig. 9a). The results of contemporary literature imply that metformin suppresses TF expression in human monocytes and decreases thrombin activity and fibrin polymerization^43^. Also Wang *et al*.^42^ reported that four-month high-dose metformin treatment led to a moderate, yet insignificant, reduction of plasma TF activity; however, they conclude that these small changes in TF activity may nevertheless be clinically relevant, as such changes in hemostatic factors have been shown to determine cardiovascular risk.

Since T2DM subjects demonstrate signs of hypercoagulability and increased plasma TF activity, the next stage of the study examined human plasma. It was found that none of the tested compounds apart from the highest concentrations of sulfonamides **7** and **8** affected the plasma TF level (Supplementary Fig. S2, Supplementary Table S2), indicating that the examined biguanides do not directly interact with this glycoprotein.

To further characterize how biguanide derivatives affect the functions of ECs and participate in vascular haemostasis, the study examined the release of von Willebrand Factor (vWF). Stimulation of HUVECs with metformin and phenformin over the entire concentration range insignificantly decreased the release of vWF from undisturbed ECs. These results are in agreement with those of Jager *et al*.^21^ who found that metformin administration in patients suffering from metabolic syndrome is associated with significant decrease in vWF level. The authors suggest that improvement in markers of endothelial functions (e.g. vWF) contributed to a decreased risk of cardiovascular disease in T2DM subjects.

Interestingly, in contrast to the beneficial effects of metformin, its derivative with a *n*-butyl chain (sulfenamide **3**) increased the concentration of vWF in cell supernatants. Among the tested compounds only sulfonamide **8** significantly reduced the amount of released vWF (61.87 ± 3.58 pg/mL at 1.0 µmol/mL vs. 83.57 ± 9.12 pg/mL, p = 0.027).

In addition, the study examined the cellular release of tissue plasminogen activator (t-PA), a serine protease synthesized in ECs and occurring in blood plasma. In the current study metformin over the entire concentration range contributed to increased release of t-PA from undisturbed ECs in *in vitro* conditions; this could be regarded beneficial since the primary role of t-PA is plasmin activation. It has also previously been^44^ confirmed that higher concentrations of t-PA (in the range of 170–1400 ng/mL) increase the initial velocity of fibrinolysis (↑ Lvo), and therefore accelerates the complete lysis of a fibrin clot. In contrast, Goya *et al*.^45^ report that the t-PA antigen is an established marker of endothelial dysfunction: particularly its elevated level, and decreased activity are associated with threefold increased risk of diabetes development. We presume that the presence of an increased level of t-PA in diabetes patients stems from the presence of fibrinolysis resistance and compensatory elevated t-PA synthesis. In our previous study^18^, we reported that metformin does not affect *in vitro* the activity of plasmin, thus the final step of enzymatic cleavage of fibrin is not disturbed. In addition, a review of current literature does only returns not give the exact answer regarding the influence of metformin on the t-PA levels in both diabetic and non-diabetic patients since the studies report conflicting effects. For instance, Serdyńska-Szustak *et al*.^46^ reported that metformin administration to women suffering from Polycystic Ovary Syndrome (PCOS) does not lead to the alterations in t-PA and vWF level. On the other hand, Jager *et al*.^21^ reported that long-term metformin treatment of diabetes subjects, was associated with reduction of plasma vWF, sVCAM-1, t-PA, and PAI-1, compared to controls.

The next purpose of this study was to assess the effects of biguanides on the expression of the ICAM-1 adhesion molecule, expressed in ECs^47^. It is well known that ICAM-1 facilitates neutrophils infiltration, and plays a central role in inflammatory and immune response^48^. Liu *et al*.^49^ highlight that metformin might down-regulate ICAM-1 in an AMPK-dependent manner, which could prevent ischemia-induced brain injury by alleviating neutrofil infiltration. The authors state that the combination of decreased ICAM-1 expression and decreased cell permeability may have the potential to be useful in the clinical treatment of ischemic stroke. Similarly, Zhang *et al*.^47^ claim that metformin down-regulates ICAM-1, blocks neutrophil infiltration and attenuates blood-spinal cord barrier (BSCB) premeability after spinal cord injury (SCI) in rats.

In turn, Germeyer *et al*.^50^ showed that metformin does not significantly alter the expression of ICAM mRNA in different cellular model. Our present findings indicate that metformin at 0.06 and 0.3 µmol/mL did not significantly affect the expression of ICAM-1 on the surface of HUVECs in comparison to unstimulated cells; nor was any effect observed in the case of phenformin. When stimulated with other sulfenamides and sulfonamides (apart from **7** and **8**; Fig. 10), HUVECs enhanced ICAM-1 expression, thus potentially increasing the accessibility of the smooth musce layer for immune cells. The effects of the tested biguanides on above-mentioned endothelial parameters are presented in Figure S4 (Supporting materials).

### Platelets thrombus formation and blood coagulation

Since the patients with T2DM present an impaired balance between the process of coagulation and fibrinolysis^18^, the influence of metformin and its derivatives on thrombogenesis was measured under a blood flow state using T-TAS, a novel and easy to use microchip-based flow chamber system. In the present study, we used PL-chips, which can assess thrombogenicity associated with platelets, and AR-chips, which determine thrombogenicity under the condition of platelets and plasma coagulation factors. It was previously demonstrated that T-TAS might be a useful tool in monitoring the anticoagulant effects of edoxaban, a non–vitamin K oral anticoagulant as well as predicting the respective risk of bleeding complications in atrial fibrillation patients^51^.

This is the first study to describe the direct effect of metformin on platelet thrombus formation using the T-TAS method. In this study, both oclussion time (OT) and clotting time (CT) were found to be significantly prolonged by metformin at concentration of 0.3 µmol/mL. We presume that these properties may result from the effects of the drug on platelets activation and aggregation since it is known to prevent platelets aggregation^52^ and decrease maximum platelets aggregation induced by adenosine diphosphate (ADP) *in vitro*^53^. The studies using AR chips revealed that metformin does not exert any effects on the total platelet and plasma thrombogenicity since no changes in measured parameters (T_10_, OT_30_, T_10–80_ and AUC_30_) were recorded. These results are in agreement with those obtained in our previous experiments^16,18^. The clot formation and lysis test (CL test) found that metformin does not change the overall potential of clot formation and fibrinolysis (CL_AUC_ constant) nor the kinetic parameters of the process of clot formation and fibrinolysis, including the initial velocity of clot formation (Fvo constant) and initial clot fibrinolysis velocity (Lvo constant). We also reported that metformin does not affect either the extrinsic or the intrinsic coagulation pathway since Prothrombin Time (PT) and Partially Activated Thromboplastic Time (APTT) were comparable to control values^16^. In addition, metformin does not influence the activity of factor X: the first member of common coagulation pathway converting prothrombin to thrombin^18^. Unlike metformin, sulfonamide **8** with *o*-nitro benzene substituent was found to prolong all parameters (↑ T_10_, ↑ OT, ↑ CT) and decrease AUC in platelets-dependent thrombus formation studies using PL chips. Furthermore, compound **8** exhibited advantageous anti-coagulant properties in fibrin-rich platelets thrombus formation studies manifested by prolonged T_10_, OT_30_, T_10–80_ and reduced AUC_30_. These results might be explained by decreased overall potential of clot formation and fibrinolysis (↓ CL_AUC_), decreased maximum clotting (↓ Fmax) and initial velocity of clot formation (↓ Fvo) in the CL test, as well as prolonged PT and APTT parameters^17^. In addition, other mechanisms including decreased activity of coagulation factor X and reduced amidolytic activity of thrombin^18^ might account for the anti-coagulation properties obtained using T-TAS. Oclussion time (OT) and clotting time (CT) were prolonged, with a simultaneous decrease in the area under the curve (AUC) observed for most examined sulfenamides and sulfonamides; therefore further studies based on *inter alia* platelets aggregation and adhesion, are needed to elucidate these favourable anti-coagulant properties. The effects of the tested biguanides on blood coagulation are summarized in Figure S5 (Supporting materials).

## Conclusions

Within this paper we evaluated the effects of metformin, and its sulfenamide and sulfonamide analogues on a few markers of endothelial function and blood coagulation. We reported that metformin, besides its established activity in lowering blood glucose, contributes to a slight increase in endothelial cell and smooth muscle cells viability and integrity. Apoptosis assay confirmed that metformin has beneficial effects on ECs since the decreased percentage of apoptotic cells was recorded. Metformin was also found to prolong the process of platelets thrombus formation, suggesting that this drug affects platelets activation and aggregation.

Examined sulfenamide and sulfonamide derivatives of metformin presented a varied effects on ECs function. For instance, the length of the alkyl chain in sulfenamides determines their properties towards ECs. Sulfenamide **3** with *n*-butyl alkyl chain, similarly to metformin, did not contribute to the changes in endothelial integrity at any of the analyzed time-points over the entire concentration range, nor ECs viability using apoptosis test (Fig. S6, Supplementary materials). Additionally, the anti-coagulation properties of compound **3** were confirmed by prolonged platelet-dependent thrombus formation and fibrin-rich platelet thrombus formation. Our results indicate that also 4-nitro-benzenesulfonamide (**7**) and 2-nitro-benzenesulfonamide (**8**) exhibit anticoagulant properties manifested by decreased vWF release and prolonged OT, CT and OT_80_ using T-TAS.

In conclusion, chemical modification of metformin scaffold into sulfenamides with different alkyl substituents and sulfonamides containing a nitro group in the aromatic ring contributes to the obtaining of potential agents with more clearly marked anti-coagulant properties than their parent drug, metformin. Therefore, these findings provide the direct evidence that new drug design strategy including modification of metformin scaffold into sulfonamides was succesfully developed, and might be regarded as a good starting point for the design and synthesis of novel biguanide-based compounds with anticoagulant properties and valuable features regarding endothelial function.

## Materials and Methods

### Materials

All the reagents used within this study are presented in detail in Supplementary materials.

### Preparation of blood and plasma samples

All experiments using human blood were performed in accordance with Polish guidelines and according to the studies protocols which were approved by the Bioethics Committee of the Medical University of Lodz (Medical University of Lodz, Poland). Approvals of the Bioethics Committee no. RNN/27/18/KE and RNN/350/18/KE were obtained for the studies on the TF quantification, and the coagulation measurements using T-TAS system, respectively. The experiments, and applied methods were carried out according to the study protocol (appendix in an application to the Bioethics Committee), and were approved by the Bioethics Committee of the Medical University of Lodz. The informed consent was signed and obtained from all subjects.

Blood samples for TF quantification were obtained from the Voivodship Specialized Hospital in Łódź, Poland (Wojewódzki Specjalistyczny Szpital im. Dr W. Biegańskiego w Łodzi), the material tested was a remnant of routine diagnostic tests intended for recycling. The blood samples for coagulation measurements were collected from healthy donors (both sex, age range 18–50, not suffering from any chronic diseases, non-smoking). The details of blood sample preparation is enclosed in Supplementary materials.

### HUVEC and AoSMC cells subculturing

HUVEC and AoSMC cells were subcultured according to the manufacturer’s (Lonza, Italy, and ScienCell Research Laboratories, US, respectively) guidelines. Cells between passages 3–5 were used in the experiments.

### Tested compounds

Within this study metformin, phenformin, five sulfenamide and three sulfonamide derivatives of metformin were examined (Fig. 1). The design and syntheses of metformin derivatives 1–8 (Fig. 1) were carried out at the University of Eastern Finland and reported previously^27–29^.

Taking into consideration our previous studies and the broad range of therapeutic plasma concentrations of metformin (0.129 to 90 mg/L^54^ which is 0.8 nmol/mL–0.6 μmol/mL) metformin and other compounds were applied in the range of 0.006–1.5 μmol/mL^16,17^.

### Cell culture in the real-time cell electric impedance sensing system (RTCA-DP)

The effects of biguanides on HUVEC viability and endothelium monolayer integrity was evaluated using an electronic method conducted on RTCA-DP system (Real-Time Cell Analyzer; Roche & ACEA Biosciences) which enables cell growth status to be evaluated in real time based on tracking electrical impedance signals. The main parameters of cell condition including viability, adhesion, morphology or barrier properties are expressed by Cell Index (CI), which is used to measure relative changes in electrical impedance^55^.

The procedure of cell culturing in RTCA DP system was previously described^55,56^. At the starting point 20 000 cells were seeded on E-16 plates in each well. Mostly, HUVEC cells reached plateau phase on the second day after seeding (the value of CI at the level of 6–8). Afterwards, the medium was extracted from each well and the solutions of tested compounds dissolved in cell culturing medium or pure medium (control) were added. After 48 hours the experiment was stopped and the values of CI were collected. The results are presented using ‘normalized cell index’ (nCI) which is calculated by division of a CI value at a certain time point by the CI value at a reference time point. The experiments were conducted in duplicates or triplicated in three independent experiments. The method was validated and the variability coefficient was counted (CV = 7.1–10.7%, depending on the measured time point, n = 6).

In the next step of our studies we determined the effects of biguanides on AoSMC viability and integrity using RTCA-DP system. The experimental procedure was the same as in the case of HUVECs, with the only difference regarding the number of seeded cells (10 000 cells per well in 16-well E-plate).

### HUVECs and AoSMC morphology

HUVEC and AoSMC cells were seeded at a density of 20 000 and 10 000 per well on 48 well plate respectively and allowed to reach 70% confluency. Then the medium alone (control) or medium with tested compounds at appropriate concentrations was added. The cells were incubated with compounds for 24 hours following by analysis of the cells morphology (100-times magnification) using an inverted microscope with phase contrast (Opta-Tech, software OptaView 7).

### HUVECs apoptosis

The cells were seeded at the density of 50 000 per well on 24 well plate and incubated for 24 hours (37 °C, 5% CO_2_). Then the medium (control) or medium with tested compounds at appropriate concentrations was added and incubated for the next 24 hours. The cells were trypsinised, trypsin neutralizing solution was added, and the cells were collected to Eppendorf tubes. After centrifugation (220 x g, five minutes) the cells were resuspended in cold cell staining buffer and washed twice with this solution. Then the cells were suspended in 100 μL of binding buffer, and solutions of propidine iodide (PI) and FITC-Annexin were added. The analysis was conducted on (FACS Canto II, Becton Dickinson) cytometer.

Annexin V(−) and PI (−) cells were considered as living cells, Annexin V (+) and PI (−) as early-apoptotic cells, Annexin V(+) and PI (+) as late-apoptotic cells, and Annexin V (−) and PI (+) as necrotic cells. The experiments were conducted in quadruplicates (n = 4). The coefficients of variation determined by triplicate assays were estimated (CV = 0.5–10.5%, depending on the measured parameter).

### Quantification of TF in HUVECs

HUVEC cells were cultured on 96-well plates and incubated until reaching 80% confluence. Afterwards 100 μL of medium was replaced by the same volume of fresh medium (control) or medium including compounds at various concentrations. The plates were incubated at 37 °C (5% CO_2_) for six hours. Following this, the cultured cells were then washed with 200 μL PBS (Biomed Lublin), lysed and solubilized with 15 mM octyl-β-D-glucopyranoside (Abcam) at 37 °C for 15–20 minutes. Cell lysates were collected into eppendorf tubes and stored for approximately one week at −20 °C until analysis.

The quantification of TF in HUVECs lysates was conducted using human tissue factor (TF) ELISA kit (Abcam, US). The coefficients of variation for the applied method were determined by two independent assays conducted in quadruplicate (CV = 4.2%). The details of the experimental procedure are described in Supplementary materials.

### Von Willebrand Factor release from HUVECs

HUVEC cells were cultured for 24 hours (37 °C, 5% CO_2_) on 48-well plates at a density of 20 000 cells per well. The next day, the medium was exchanged for 100 μL medium containing test compounds, for the study group, or 100 μL fresh medium, for the control samples. The plates were further incubated at 37 °C (5% CO_2_) for 24 hours. Following this, the cell supernatants were collected into Eppendorf tubes, centrifuged (5 min, 220 × g) and stored at −20 °C until analysis.

The quantification of vWF released from HUVECs was carried out using human vWF ELISA assay (Biorbyt, United Kingdom). The coefficients of variation for this method were determined by three independent assays conducted in quadruplicate (CV = 9.3%). The details of the experimental procedure are described in Supplementary materials.

### Tissue Plasminogen Activator (t-PA) release from HUVECs

For quantitative measurements of t-PA, HUVEC cells were prepared in a similar way to previously described studies. The cells were seeded on 96-well plates at a density of 10 000 cells per well and allowed to become confluent (37 °C, 5% CO_2_). After 24 hours of incubation, the medium was changed in control wells and medium containing tested compounds at appropriate concentration was added. The plates were further incubated at 37 °C (5% CO_2_) for the next 24 hours. The cell supernatants were then collected into Eppendorf tubes, centrifuged (5 min, 220 x g), diluted three-fold, and stored at −20 °C until analysis.

The quantification of t-PA in HUVECs supernatants was conducted using a human t-PA ELISA kit (Abcam, US). The coefficients of variation for this method was determined by two independent assays conducted in quadruplicate (CV = 3.9%). The details of the experimental procedure are described in Supplementary materials.

### ICAM expression on HUVECs surface

The cell samples were prepared according the procedure described for apoptosis tests. The cells were trypsinised, trypsin neutralizing solution was added; following this, the cells were transferred to Eppendorf tubes and centrifuged (220 x g, five minutes). The cell pellets were then suspended in cold cell staining buffer and centrifuged (220 x g, five minutes). Then the buffer was added again (100 μL), and solution of PE anti-human CD54 antibody was added. Controls were set up using PE mouse IgG1 kappa isotype. The results are presented as the percentage of the total cells bound with CD54 antibody (the number of replicates of the experiments was between 3–6). The coefficients of variation for the method was counted (CV = 7.9%, n = 6).

### Thrombus formation studies

Thrombus formation under flow conditions was evaluated using the Total Thrombus-Formation Analysis System (T-TAS; Fujimori Kogyo, Tokyo, Japan)^57^, which is a simple procedure for analyzing the process of thrombus formation in blood. A collagen coated microchip (Platelet Chip; PL-Chip) was used to evaluate platelet-dependent thrombus formation. A blood sample (480 μL) anticoagulated with hirudin was transfered to the PL-chip at a flow rate of 24 μL/min, which corresponds to an initial wall shear force of 2000 s^−1^. During the blood flow, platelets aggregate on the surface of collagen, and the microchip capillaries become occluded. Measurements were taken of changes in flow pressure caused by this capillary occlusion to follow the process of thrombus formation. The following parameters were counted: T_10_ – defined as the onset of platelet thrombus formation and is counted as the time for the flow pressure to increase to 10 kPa from baseline due to a partial occlusion of the capillary; OT - occlusion time, which is the time for the flow pressure to increase by 60 kPa from baseline owing to near complete occlusion of the capillary by platelets thrombus; Clotting time (CT) – the difference between OT and T_10_; AUC_10_ - area under the flow pressure curve for 10 minutes, it gives the information on total thrombogenicity as well as the growth and stability of the formed thrombi. The studies were conducted in three independent experiments on blood samples from different donors.

The second type of chip, a collagen and thromboplastin coated microchip (Atheroma Chip; AR-Chip) was used for analysis of fibrin-rich platelet thrombus formation at a shear rate of 600 s^−1^ (10 μL/min)^58^. For measurements with the AR-chip, citrated blood (480 μL) was mixed with 20 μL of Ca^2+^-corn trypsin inhibitor (CTI) (0.3 M CaCl_2_ and 1.25 mg/mL CTI) and immediately applied to the AR microchip. As the process of thrombus formation proceeds, the capillary is gradually occluded, and the flow pressure increases. Similarly to PL microchips, there are four parameters which characterises the coagulation process: T_10_ (time to reach 10 kPa) is the onset of white thrombus formation (WTF); T_80_ (time to reach 80 kPa) is defined as the complete occlusion of capillary due to thrombus formation, T_10–80_ (time from 10 to 80 kPa) is the interval between T_10_ and OT; AUC_30_ constitutes the area under the flow pressure curve for 30 minutes, and is used to quantify thrombogenicity of the blood sample^51,59,60^. The experiments were conducted in three independent experiments on blood samples from different donors.

The intra-individual coefficients of variation (CV, %) determined from different individuals were as follows: for PL-chips 9.7–18.5%, n = 7, for AR-chips 1.0–11.5%, n = 3, depending on the measured parameter.

### Statistical analysis

All statistical calculations were performed using Statistica 12.0 (StatSoft) and Prism 5 (GraphPad). The results are presented as the mean ± standard deviation (SD) for variables with a normal distribution of values. The normal distribution of continuous variables was verified with the Shapiro-Wilk test, whereas Levene’s test was performed to test the homogeneity of variances. All variables with a normal distribution were compared to respective controls using the paired t-test, while one or two-way Anova and subsequent *post hoc* tests were used for intergroup comparisons. The variables with non-normal distributions were compared using the Wilcoxon signed rank test. The results of all the tests were considered significant at *p*-values lower than 0.05.

## Supplementary information

Supplementary information

## Data Availability

The data collected within this study is available on request.

## References

[1] Grzybowska M, Bober J, Olszewska M (2011). Metformin - mechanisms of action and use for the treatment of type 2 diabetes mellitus. Postep. Hig Med Dosw.

[2] Bailey CJ (2017). Metformin: historical overview. Diabetologia.

[3] Markowicz-Piasecka M, Huttunen KM, Mateusiak L, Mikiciuk-Olasik E, Sikora J (2017). Is Metformin a Perfect Drug? Updates in Pharmacokinetics and Pharmacodynamics. Curr. Pharm. Des..

[4] Kooy, A., Zeeuw, D. D. E., Stehouwer, C. D. A. & Gansevoort, R. T. The effect of metformin on blood pressure, plasma cholesterol and triglycerides in type 2 diabetes mellitus: a systematic review. 1–14 (2004). 10.1111/j.1365-2796.2004.01328.x 15189360

[5] Markowicz-Piasecka M (2017). Metformin – a Future Therapy for Neurodegenerative Diseases: Theme: Drug Discovery, Development and Delivery in Alzheimer’s Disease Guest Editor: Davide Brambilla. Pharm. Res..

[6] Markowicz-Piasecka M, Huttunen KM, Mikiciuk-Olasik E, Mateusiak Ł, Sikora J (2018). Metformin-from anti-diabetic drug to anti-cancer drug. Acta Pol. Pharm. - Drug Res..

[7] UK Prospective Diabetes Study (UKPDS) Group. Effect of intensive blood-glucose control with metformin on complications in overweight patients with type 2 diabetes (UKPDS 34). *Lancet* 854–865 (1998). 9742977

[8] Holman RR, Paul SK, Bethel MA, Matthews DR (2008). N. H. 10-year follow-up of intensive glucose control in type 2 diabetes. N. Engl. J. Med..

[9] Hong J (2012). Effects of metformin versus glipizide on cardiovascular outcomes in patients with type 2 diabetes and coronary artery disease. Diabetes Care.

[10] Yang X, Xu Z, Zhang C, Cai Z, Zhang J (2017). Metformin, beyond an insulin sensitizer, targeting heart and pancreatic β cells. Biochim. Biophys. Acta - Mol. Basis Dis..

[11] Kinsara AJ, Ismail YM (2018). Metformin in heart failure patients. Indian Heart J..

[12] Chi C, Snaith J, Gunton JE (2017). Diabetes Medications and Cardiovascular Outcomes in Type 2 Diabetes. Hear. Lung Circ..

[13] Nesti L, Natali A (2017). Metformin effects on the heart and the cardiovascular system: A review of experimental and clinical data. Nutr. Metab. Cardiovasc. Dis..

[14] Yang Q (2018). Metformin ameliorates the progression of atherosclerosis via suppressing macrophage infiltration and inflammatory responses in rabbits. Life Sci..

[15] William T (2002). Effect of Combination Glipizide GITS/Metformin on Fibrinolytic and Metabolic Parameters in Poorly Controlled Type 2 Diabetic Subjects. Diabetes Care.

[16] Markowicz-Piasecka M, Sikora J, Mateusiak Ł, Mikiciuk-Olasik E, Huttunen KM (2017). New prodrugs of metformin do not influence the overall haemostasis potential and integrity of the erythrocyte membrane. Eur. J. Pharmacol..

[17] Markowicz-Piasecka M, Huttunen KM, Mikiciuk-Olasik E, Sikora J (2018). Biocompatible sulfenamide and sulfonamide derivatives of metformin can exert beneficial effects on plasma haemostasis. Chem. Biol. Interact..

[18] Markowicz-Piasecka M, Huttunen KM, Mateusiak Ł, Mikiciuk-Olasik E, Sikora J (2018). Sulfenamide and sulfonamide derivatives of metformin can exert anticoagulant and profibrinolytic properties. Chem. Biol. Interact..

[19] Duan Quanlu, Song Ping, Ding Ye, Zou Ming-Hui (2017). Activation of AMP-activated protein kinase by metformin ablates angiotensin II-induced endoplasmic reticulum stress and hypertension in mice in vivo. British Journal of Pharmacology.

[20] Agard C (2009). Protective role of the antidiabetic drug metformin against chronic experimental pulmonary hypertension. Br. J. Pharmacol..

[21] de Jager J (2014). Long-term effects of metformin on endothelial function in type 2 diabetes: A randomized controlled trial. J. Intern. Med..

[22] Venna VR, Li J, Hammond MD, Mancini NS, Mccullough LD (2014). Chronic metformin treatment improves post-stroke angiogenesis and recovery after experimental stroke. Eur. J. Neurosci..

[23] Kinaan M, Ding H, Triggle CR (2015). Metformin: An Old Drug for the Treatment of Diabetes but a New Drug for the Protection of the Endothelium. Med. Princ. Pract..

[24] Vitale C (2005). Metformin improves endothelial function in patients with metabolic syndrome. J. Intern. Med..

[25] Tabit CE, Chung WB, Hamburg NM, Vita JA (2010). Endothelial dysfunction in diabetes mellitus: Molecular mechanisms and clinical implications. Rev. Endocr. Metab. Disord..

[26] Kadoglou NP, Tsanikidis HKA (2010). Effects of rosiglitazone and metformin treatment on apelin, visfatin, and ghrelin levels in patients with type 2 diabetes mellitus. Metabolism.

[27] Huttunen KM (2009). The first bioreversible prodrug of metformin with improved lipophilicity and enhanced intestinal absorption. J. Med. Chem..

[28] Huttunen KM, Leppänen J, Laine K, Vepsäläinen J, Rautio J (2013). Convenient microwave-assisted synthesis of lipophilic sulfenamide prodrugs of metformin. Eur. J. Pharm. Sci..

[29] Rautio J, Vernerová M, Aufderhaar I, Huttunen KM (2014). Glutathione-S-transferase selective release of metformin from its sulfonamide prodrug. Bioorganic Med. Chem. Lett..

[30] Gorzelak-Pabis P (2017). Endothelial integrity may be regulated by a specific antigen via an IgE-mediated mechanism. Postepy Hig. Med. Dosw. (Online).

[31] He L, Wondisford FE (2015). Essay Metformin Action: Concentrations Matter. Cell Metab..

[32] Esfahanian N (2012). Effect of metformin on the proliferation, migration, and MMP-2 and −9 expression of human umbilical vein endothelial cells. Mol. Med. Rep..

[33] Arunachalam G, Samuel SM, Marei I, Ding H, Triggle CR (2014). Metformin modulates hyperglycaemia-induced endothelial senescence and apoptosis through SIRT1. Br. J. Pharmacol..

[34] Majithiya JB, Balaraman R (2006). Metformin reduces blood pressure and restores endothelial function in aorta of streptozotocin-induced diabetic rats. Life Sci..

[35] Li, L., Mamputu, J. & Wiernsperger, N. Signaling Pathways Involved in Human Vascular Smooth Muscle Cell Proliferation and Matrix Metalloproteinase-2 Expression Induced by Leptin Inhibitory Effect of Metformin. 54, 2227–2234 (2005). 10.2337/diabetes.54.7.2227 15983226

[36] Hao, B., Xiao, Y., Song, F., Long, X. & Huang, J. Metformin-induced activation of AMPK inhibits the proliferation and migration of human aortic smooth muscle cells through upregulation of p53 and IFI16. 1365–1376, 10.3892/ijmm.2017.3346 (2018). 10.3892/ijmm.2017.3346 PMC5819901 29286156

[37] Bakhashab S (2018). Proangiogenic effect of metformin in endothelial cells is via upregulation of VEGFR1/2 and their signaling under hyperglycemia-hypoxia. Int. J. Mol. Sci..

[38] Detaille D (2005). Metformin Prevents High-Glucose-Induced Endothelial Cell Death Through a Mitochondrial Permeability Transition-Dependent Process. Diabetes.

[39] Mu Q (2018). Metformin inhibits proliferation and cytotoxicity and induces apoptosis via AMPK pathway in CD19-chimeric antigen receptor-modified T cells. Onco. Targets. Ther..

[40] Queiroz Eveline A. I. F., Puukila Stephanie, Eichler Rosangela, Sampaio Sandra C., Forsyth Heidi L., Lees Simon J., Barbosa Aneli M., Dekker Robert F. H., Fortes Zuleica B., Khaper Neelam (2014). Metformin Induces Apoptosis and Cell Cycle Arrest Mediated by Oxidative Stress, AMPK and FOXO3a in MCF-7 Breast Cancer Cells. PLoS ONE.

[41] Sahra IB, Laurent K, Loubat A, Giorgetti-Peraldi SCP (2006). The antidiabetic drug metformin exerts an antitumoral effect *in vitro* and *in vivo* through a decrease of cyclin D1 level. Oncogene.

[42] Wang J, Ciaraldi TP, Samad F (2015). Tissue factor expression in obese type 2 diabetic subjects and its regulation by antidiabetic agents. J Obes.

[43] Standeven KF (2002). The effect of dimethylbiguanide on thrombin activity, FXIII activation, fibrin polymerization, and fibrin clot formation. Diabetes.

[44] Kostka B, Para J, Sikora J (2007). A multiparameter test of clot formation and fibrinolysis for *in-vitro* drug screening. Blood Coagul. Fibrinolysis.

[45] Goya S, Peter H (2008). Tissue Plasminogen Activator, von Willebrand Factor, and Risk of Type 2 Diabetes in Older Men. Diabetes Care.

[46] Serdyńska Szuster M, Banaszewska B, Spaczyński R, Pawelczyk L (2011). Wpływ leczenia metformina na wykładniki zaburzeń krzepniecia u kobiet z zespołem policystycznych jajników i insulinoopornościa. Ginekol. Pol..

[47] Zhang D (2017). Metformin ameliorates BSCB disruption by inhibiting neutrophil infiltration and MMP-9 expression but not direct TJ proteins expression regulation. J. Cell. Mol. Med..

[48] Hadad N, Tuval L, Elgazar-Carmom V, Levy R, Levy R (2011). Endothelial ICAM-1 Protein Induction Is Regulated by Cytosolic Phospholipase A2 via Both NF- B and CREB Transcription Factors. J. Immunol..

[49] Liu Y (2014). Metformin attenuates blood-brain barrier disruption in mice following middle cerebral artery occlusion. J Neuroinflammation..

[50] Germeyer A (2011). Metformin modulates IL-8, IL-1β, ICAM and IGFBP-1 expression in human endometrial stromal cells. Reprod. Biomed. Online.

[51] Sueta D (2015). A novel quantitative assessment of whole blood thrombogenicity in patients treated with a non-vitamin K oral anticoagulant. Int. J. Cardiol..

[52] Colwell J (2001). Treatment for the procoagulant state in type 2 diabetes. Endocrinol. Metab. Clin. North Am..

[53] Gin H, Freyburger G, Boisseau MAJ (1989). Study of the effect of metformin on platelet aggregation in insulin-dependent diabetics. Diabetes Res Clin Pr..

[54] Kajbaf F, De Broe ME, Lalau JD (2016). Therapeutic Concentrations of Metformin: A Systematic Review. Clin. Pharmacokinet..

[55] Chalubinski M (2013). The effect of 7-ketocholesterol and 25-hydroxycholesterol on the integrity of the human aortic endothelial and intestinal epithelial barriers. Inflamm. Res..

[56] Markowicz-Piasecka M (2014). Studies towards biocompatibility of PAMAM dendrimers - Overall hemostasis potential and integrity of the human aortic endothelial barrier. Int. J. Pharm..

[57] Ogawa S (2011). A comparative study of prothrombin complex concentrates and freshfrozen plasma for warfarin reversal under static and flow conditions. Thromb. Haemost..

[58] Hosokawa K (2011). A novel automated microchip flow-chamber system to quantitatively evaluate thrombus formation and antithrombotic agents under blood flow conditions. J. Thromb. Haemost..

[59] Hosokawa K (2012). A microchip flow-chamber system for quantitative assessment of the platelet thrombus formation process. Microvasc. Res..

[60] Ågren, A. et al. Monitoring of coagulation factor therapy in patients with von Willebrand disease type 3 using a microchip flow chamber system. 1–8 (2017). 10.1160/TH16-06-0430 27761577

