# Supplementary material for: Sulfenamide and Sulfonamide Derivatives of Metformin – A New Option to Improve Endothelial Function and Plasma Haemostasis
Source: Sci Rep. 2019 Apr 25;9:6573. doi: 10.1038/s41598-019-43083-z (PMC6484023; doi:10.1038/s41598-019-43083-z)
Supplement: Supplementary file 1 — Supplementary information [file 41598_2019_43083_MOESM1_ESM.pdf]

SUPPLEMENTARY MATERIAL TO THE PUBLICATION:

Sulfenamide and sulfonamide derivatives of metformin – a new option to improve endothelial function and plasma haemostasis

Authors: Magdalena Markowicz-Piasecka, Kristiina M. Huttunen, Marlena Broncel, Joanna Sikora

MATERIALS AND METHODS

2. 1. Materials

Human umbilical vein endothelial cells (HUVEC) (Lonza, Italy) were used in this study. Medium EGM-2 - medium + bullet kit (Lonza, Clonetics, Italy), trypsin-EDTA – 0.05% solution (Sigma Aldrich, Germany), trypsin neutralizing solution (Lonza, Italy), HEPES buffered saline solution (Lonza, Italy) were used for cell culturing.

Aortal Smooth Muscle Cells (ScienCell Research Laboratories, US) were cultured according to the manufacturer guidelines. SMC medium consisting of 500 ml of basal medium, 10 ml of fetal bovine serum (FBS, ScienCell Research Laboratories, US), 5 ml of smooth muscle cell growth supplement (SMCGS, ScienCell Research Laboratories, US), and 5 ml of penicillin/streptomycin solution (ScienCell Research Laboratories, US) were used for cell culturing.

E-Plate 16 View (Roche & ACEA Biosciences) and phosphate buffered saline (PBS, Biomed Lublin, Poland) were used for evaluation of viability of HUVECs in the real-time cell electric impedance sensing system. Apoptosis was examined using FITC Annexin V Apoptosis Detection Kit with PI (Biolegend, United Kingdom) and cell staining buffer (Biolegend, United Kingdom). For the measurements of tissue factor (TF) activity human tissue factor ELISA Kit (CD142) (Abcam, US) was used. The quantity of von Willebrand factor in cell culture supernatants was estimated by Human Von Willebrand Factor ELISA Kit (Biorbyt, United Kingdom). The concentration of t-PA in HUVECs supernatants was performed using human tissue plasminogen activatior ELISA kit (Abcam, US). To assess ICAM-1 surface expression on HUVECs, PE anti-human CD54 Antibody (BioLegend, United Kingdom) and PE Mouse IgG1, kappa Isotype Ctrl FC (BioLegend, United Kingdom) were used.

2. 2. Blood samples preparation for T-TAS thrombogenicity measurements

The blood was collected from the antecubital vein with a 21-gauge butterfly needle into the following tubes: a hirudin-containing blood sampling tube (MP0600 (Verum Diagnostia), final hirudin concentration 25 μg/mL) for platelets thrombus formation and vacuum tubes containing

3.2% buffered sodium citrate (1:9) (Becton Dickinson) for platelets and fibrin clot formation. The blood samples were stored at room temperature up to 3 hours before the measurements.

2. 8. Quantification of TF in HUVECs

Standard samples of TF at the concentration 0 – 400 pg/mL or test samples (50 μL) were added on a 96-well plate precoated with TF specific antibody and incubated for 2 hours. The plate was washed five times with washing buffer (200 μL), and a TF specific biotinylated detection antibody was added (50 μL), following by 2 hours incubation and another set of washing. Then streptavidin-peroxidase conjugate was added and unbound conjugates were washed away with wash buffer. The next step included addition of chromogenic substrate to visualize streptavidin-peroxidase enzymatic reaction. After 10 minutes incubation at room temperature the reaction was stopped by addition of acidic stop solution (50 μL), and the absorbance was read at 450 nm (iMARK, Bio-Rad). The density of yellow colour is directly proportional to the amount of TF captured in plate. The amount of TF in HUVECs was calculated using calibration curve (R^2 = 0.964); the test was conducted in quadruplicate (n = 4).

To further elucidate whether tested biguanides might directly interact with TF additional experiments using human plasma were conducted. Compounds were incubated for 6 hours with twice diluted citrate plasma, and afterwards the samples (50 μL) were analysed using above-described method.

2. 9. Von Willebrand Factor release from HUVECs

Standard samples of vWF (range 0 – 1500 pg/mL) or test samples in a volume of 100 μL were added on a 96-well plates precoated with specific vWF antibody and incubated for 90 minutes at room temperature with gentle shaking. Then the plates were washed with provided wash buffer working solution (200 μL), and appropriately diluted biotin-labeled detection antibody working solution was added (100 μL), followed by 1 hour incubation at 37 °C and another set of washing. Streptavidin-HRP working solution was added into each well, incubated for another 45 minutes at 37 °C, and washed once again. 100 μL of chromogenic substrate was added, and the colour turned into blue. The plates were incubated for 30 minutes at 37 °C in the dark, and the acidic stop solution (100 μL) was added. The measurements were taken at 450 nm using microplate reader (iMARK, Bio-Rad). The amount of vWF released from HUVECs was calculated using calibration curve (R^2 = 0.969); the number of replicates in the test was 4-6 (n = 4-6).

2. 10. Tissue Plasminogen Activator (t-PA) release from HUVECs

A t-PA specific antibody has been precoated onto 96-well plates and blocked. Standard samples (0 – 1 ng/mL) of t-PA or test samples in a volume of 50 μL were added and incubated for 2

hours. The plate was washed five times with washing buffer (200 μL), and subsequently a t-PA specific biotinylated detection antibody was added followed by washing with wash buffer. Then Streptavidin-Peroxidase Conjugate was added and unbound conjugates were washed away with wash buffer. Chromogenic substrate (TMB; 3,3',5,5'-tetramethylbenzidine) was used to visualize enzymatic reaction (blue color product). The last step incuded addition of acidic stop solution and immediate absorbance measurements at the wavelength of 450 nm (iMARK, Bio-Rad). The density of yellow coloration was proportional to the amount of t-PA captured in plate. The amount of t-PA in HUVECs supernatant was calculated using calibration curve (R^2 = 0.983); the number of replicates in the test was 4 (n = 4).

RESULTS

Supplementary Table S1. The effects of biguanides on the integrity of human endothelial cells

**Metformin**	Control	0.006 μmol/mL	0.06 μmol/mL	0.3 μmol/mL	1.5 μmol/mL
**3 h**	1.12 ± 0.16	1.34 ± 0.13	1.27 ± 0.15	1.25 ± 0.15	1.23 ± 0.16
**6 h**	1.06 ± 0.10	1.12 ± 0.08	1.10 ± 0.09	1.09 ± 0.09	1.08 ± 0.09
**12 h**	1.07 ± 0.09	1.11 ± 0.06	1.06 ± 0.09	1.04 ± 0.09	1.04 ± 0.09
**24 h**	0.95 ± 0.07	0.99 ± 0.04	0.97 ± 0.06	0.94 ± 0.05	0.95 ± 0.08
**36 h**	0.84 ± 0.10	0.87 ± 0.10	0.87 ± 0.05	0.85 ± 0.05	0.85 ± 0.09
**Phenformin**	Control	0.006 μmol/mL	0.06 μmol/mL	0.3 μmol/mL	1.5 μmol/mL
**3 h**	1.25 ± 0.16	1.33 ± 0.13	1.30 ± 0.14	1.20 ± 0.10	**1.06 ± 0.07***
**6 h**	1.12 ± 0.10	1.13 ± 0.08	1.12 ± 0.10	1.04 ± 0.06	**0.83 ± 0.09*****
**12 h**	1.07 ± 0.09	1.12 ± 0.04	1.10 ± 0.09	1.02 ± 0.04	**0.73 ± 0.16*****
**24 h**	0.95 ± 0.07	1.00 ± 0.04	1.01 ± 0.01	0.98 ± 0.04	**0.48 ± 0.28*****
**36 h**	0.83 ± 0.10	0.89 ± 0.10	0.93 ± 0.05	0.94 ± 0.09	**0.32 ± 0.28*****
**Comp. 1**	Control	0.006 μmol/mL	0.06 μmol/mL	0.3 μmol/mL	1.5 μmol/mL
**3 h**	1.31 ± 0.20	1.15 ± 0.12	**0.07 ± 0.02*****	**0.00 ± 0.01*****	**0.00 ± 0.01*****
**6 h**	1.16 ± 0.09	**1.03 ± 0.07***	**0.06 ± 0.02*****	**0.00 ± 0.01*****	**0.00 ± 0.01*****
**12 h**	1.15 ± 0.07	**1.00 ± 0.05***	**0.03 ± 0.01*****	**0.00 ± 0.01*****	**0.00 ± 0.01*****
**24 h**	1.02 ± 0.09	**0.74 ± 0.04*****	**0.01 ± 0.00*****	**0.01 ± 0.01*****	**0.00 ± 0.00*****
**36 h**	0.87 ± 0.17	**0.55 ± 0.05*****	**0.01 ± 0.01*****	**0.02 ± 0.01*****	**0.00 ± 0.00*****
**Comp. 2**	Control	0.006 μmol/mL	0.06 μmol/mL	0.3 μmol/mL	1.5 μmol/mL
**3 h**	1.27 ± 0.17	1.20 ± 0.14	**1.10 ± 0.07***	**1.02 ± 0.13*****	**0.01 ± 0.01*****
**6 h**	1.12 ± 0.09	1.01 ± 0.07	1.02 ± 0.04	1.01 ± 0.13	**0.01 ± 0.01*****
**12 h**	1.11 ± 0.09	1.14 ± 0.10	1.04 ± 0.03	0.98 ± 0.10	**0.01 ± 0.01*****
**24 h**	0.99 ± 0.09	1.00 ± 0.03	1.03 ± 0.02	1.00 ± 0.12	**0.01 ± 0.01*****
**36 h**	0.86 ± 0.13	0.87 ± 0.09	0.98 ± 0.06	1.01 ± 0.13	**0.01 ± 0.01*****
**Comp. 3**	Control	0.006 μmol/mL	0.06 μmol/mL	0.3 μmol/mL	1.5 μmol/mL
**3 h**	1.22 ± 0.16	1.28 ± 0.11	1.26 ± 0.11	1.24 ± 0.11	1.17 ± 0.07
**6 h**	1.10 ± 0.09	1.05 ± 0.08	1.08 ± 0.07	1.07 ± 0.07	1.06 ± 0.06
**12 h**	1.18 ± 0.14	1.20 ± 0.08	1.13 ± 0.09	1.12 ± 0.09	1.02 ± 0.05
**24 h**	1.06 ± 0.11	1.04 ± 0.02	1.03 ± 0.05	1.03 ± 0.03	1.01 ± 0.03
**36 h**	0.91 ± 0.11	0.90 ± 0.08	0.91 ± 0.09	0.92 ± 0.08	0.96 ± 0.04

(HUVECs) analyzed in the RTCA-DP system.

**Comp. 4**	Control	0.006 µmol/mL	0.06 µmol/mL	0.3 µmol/mL	1.5 µmol/mL
**3 h**	1.22 ± 0.06	1.28 ± 0.19	1.19 ± 0.08	**1.09 ± 0.05****	**0.83 ± 0.07*****
**6 h**	1.06 ± 0.04	1.18 ± 0.10	1.02 ± 0.04	0.96 ± 0.06	**0.76 ± 0.11*****
**12 h**	1.07 ± 0.04	1.17 ± 0.06	1.03 ± 0.05	0.98 ± 0.02	**0.58 ± 0.05*****
**24 h**	1.01 ± 0.05	1.08 ± 0.05	0.99 ± 0.06	1.00 ± 0.02	**0.52 ± 0.03*****
**36 h**	0.91 ± 0.03	0.95 ± 0.09	0.95 ± 0.08	0.99 ± 0.03	**0.49 ± 0.03*****
**Comp. 5**	Control	0.006 µmol/mL	0.06 µmol/mL	0.3 µmol/mL	1.5 µmol/mL
**3 h**	1.21 ± 0.04	**0.88 ± 0.04****	**0.89 ± 0.12****	**0.81 ± 0.06*****	**0.60 ± 0.03*****
**6 h**	1.04 ± 0.03	0.93 ± 0.03	0.93 ± 0.09	**0.87 ± 0.05*****	**0.62 ± 0.09*****
**12 h**	1.06 ± 0.04	0.99 ± 0.02	0.98 ± 0.04	0.92 ± 0.05	**0.57 ± 0.09*****
**24 h**	1.00 ± 0.06	1.01 ± 0.02	1.03 ± 0.04	0.91 ± 0.05	**0.45 ± 0.07*****
**36 h**	0.88 ± 0.07	0.93 ± 0.03	1.01 ± 0.09	0.92 ± 0.06	**0.36 ± 0.07*****
**Comp. 6**	Control	0.006 µmol/mL	0.06 µmol/mL	0.3 µmol/mL	1.5 µmol/mL
**3 h**	1.13 ± 0.02	**0.60 ± 0.04*****	**0.69 ± 0.08*****	**0.69 ± 0.04*****	**0.08 ± 0.01*****
**6 h**	0.92 ± 0.02	**0.78 ± 0.04*****	0.88 ± 0.05	0.85 ± 0.06	**0.17 ± 0.01*****
**12 h**	0.99 ± 0.04	0.91 ± 0.03	0.91 ± 0.06	0.98 ± 0.08	**0.23 ± 0.01*****
**24 h**	1.04 ± 0.08	1.00 ± 0.04	1.10 ± 0.01	0.87 ± 0.06	**0.10 ± 0.00*****
**36 h**	1.00 ± 0.04	1.01 ± 0.02	1.06 ± 0.01	0.86 ± 0.02	**0.01 ± 0.00*****
**Comp. 7**	Control	0.006 µmol/mL	0.06 µmol/mL	0.3 µmol/mL	1.5 µmol/mL
**3 h**	1.16 ± 0.05	**0.83 ± 0.03****	**0.84 ± 0.03****	**0.72 ± 0.02*****	**0.34 ± 0.09*****
**6 h**	0.95 ± 0.06	0.86 ± 0.01	0.90 ± 0.03	**0.83 ± 0.06*****	**0.48 ± 0.06*****
**12 h**	1.01 ± 0.04	0.96 ± 0.03	0.96 ± 0.04	**0.88 ± 0.02****	**0.47 ± 0.05*****
**24 h**	1.00 ± 0.08	1.02 ± 0.07	0.99 ± 0.10	0.91 ± 0.07	**0.49 ± 0.06*****
**36 h**	0.93 ± 0.11	0.99 ± 0.04	0.95 ± 0.14	0.90 ± 0.12	**0.56 ± 0.07*****
**Comp. 8**	Control	0.006 µmol/mL	0.06 µmol/mL	0.3 µmol/mL	1.5 µmol/mL
**3 h**	1.15 ± 0.05	1.00 ± 0.06	0.92 ± 0.03	**0.78 ± 0.10****	**0.49 ± 0.30*****
**6 h**	1.05 ± 0.04	0.98 ± 0.05	0.92 ± 0.06	0.85 ± 0.05	**0.54 ± 0.18*****
**12 h**	1.14 ± 0.09	1.06 ± 0.04	0.96 ± 0.04	0.85 ± 0.03	**0.51 ± 0.21*****
**24 h**	1.10 ± 0.15	1.06 ± 0.08	0.96 ± 0.07	0.84 ± 0.09	**0.51 ± 0.22*****
**36 h**	1.11 ± 0.13	1.00 ± 0.08	0.90 ± 0.10	0.77 ± 0.17	**0.51 ± 0.24*****

The results are presented as mean ± SD, n = 6 – 8. The values given in bold represent statistically significant; * p < 0.05; ** p < 0.01; *** p < 0.001 changes versus control. The table includes the calculated normalized Cell Index values (nCI) for metformin, phenformin and 8 derivatives after selected time points (3 – 36 h) of incubation.

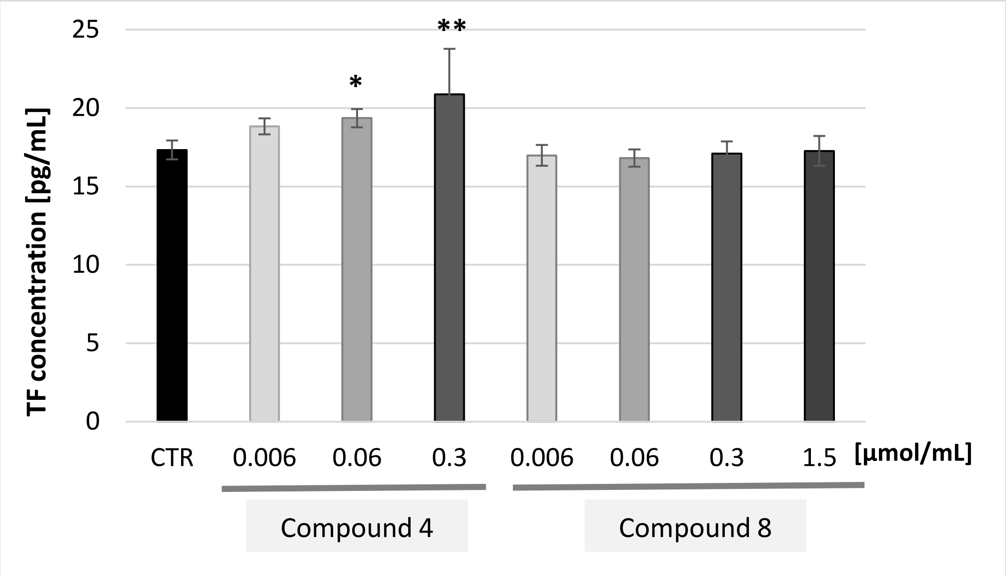

Supplementary Figure S1. The effects of sulfenamide 4 and sulfonamide 8 on the total cellular production of TF in undisturbed HUVEC cells. The results are presented as mean ± SD; n = 4; * p < 0.05, ** p < 0.01. The quantification of TF in cell lysates was conducted using immunohistochemichal ELISA test. Compound 4 at the concentration of 0.06 and 0.3 µmol/mL contributed to the significant increase in the level of intracellular TF, while compound 8 did not affect TF in HUVEC cells.

Supplementary Table S2. The effects of biguanides on human plasma tissue factor (TF) levels.

Compounds	Concentration	TF concentration [pg/mL]
Control		*135.89 ± 6.48*
**Metformin**	0.3 µmol/mL	131.87 ± 4.45
**Metformin**	1.5 µmol/mL	136.96 ± 6.73
**Phenformin**	0.06 µmol/mL	141.96 ± 4.54
**Phenformin**	0.3 µmol/mL	130.89 ± 5.07
**Compound 1**	0.006 µmol/mL	130.18 ± 10.90
**Compound 2**	0.06 µmol/mL	130.18 ± 4.94
**Compound 2**	0.3 µmol/mL	139.72 ± 7.35
**Compound 3**	0.3 µmol/mL	127.50 ± 4.76
**Compound 3**	1.5 µmol/mL	139.91 ± 10.15
**Compound 4**	0.06 µmol/mL	126.61 ± 4.54
**Compound 4**	0.3 µmol/mL	142.67 ± 8.96
**Compound 5**	0.06 µmol/mL	124.91 ± 3.93
**Compound 5**	0.3 µmol/mL	128.39 ± 8.70
**Compound 6**	0.006 µmol/mL	125.18 ± 4.65
**Compound 6**	0.06 µmol/mL	128.21 ± 4.52
**Compound 7**	0.3 µmol/mL	143.09 ± 10.78
**Compound 7**	1.5 µmol/mL	**115.00 ± 9.76***
**Compound 8**	0.3 µmol/mL	114.64 ± 10.30
**Compound 8**	1.5 µmol/mL	**100.89 ± 1.97*****

The values given in bold represent statistically significant (* p < 0.05; *** p < 0.001) changes versus control. The results are presented as mean ± SD; n = 4.

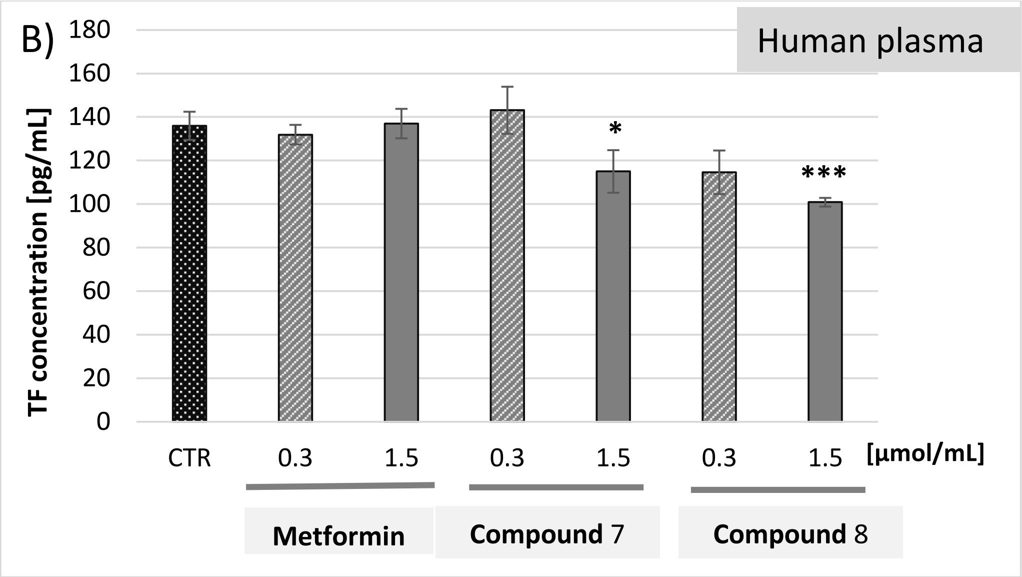

Supplementary Figure S2. The effects of metformin and sulfonamides 7 and 8 on the TF level in human plasma. The results are presented as mean ± SD; n = 4. Metformin did not affect the plasma TF while compounds 7 and 8 at the highest concentration tested significantly reduced TF level (* $p < 0.05$; *** $p < 0.001$).

Supplementary Table S3. The release of von Willebrand Factor from HUVEC cells in the presence of biguanides.

Compounds	Concentration	vWF concentration [pg/mL]
Control		*86.08* ± 8.23
**Compound 3**	0.006 µmol/mL	83.43 ± 10.13
**Compound 3**	0.06 µmol/mL	85.43 ± 12.28
**Compound 3**	0.3 µmol/mL	86.41 ± 6.05
**Compound 3**	1.5 µmol/mL	**97.28 ± 4.65***
Control		*83.57 ± 9.12*
**Compound 4**	0.006 µmol/mL	79.37 ± 3.83
**Compound 4**	0.06 µmol/mL	80.27 ± 3.06
**Compound 4**	0.3 µmol/mL	78.30 ± 8.16
**Compound 4**	1.0 µmol/mL	92.32 ± 3.89
**Compound 5**	0.006 µmol/mL	74.02 ± 4.27
**Compound 5**	0.06 µmol/mL	76.51 ± 8.19
**Compound 5**	0.3 µmol/mL	76.43 ± 8.32
**Compound 5**	1.0 µmol/mL	**98.04 ± 9.48***
**Compound 6**	0.006 µmol/mL	79.64 ± 2.26
**Compound 6**	0.06 µmol/mL	71.87 ± 3.95
**Compound 6**	0.3 µmol/mL	74.29 ± 3.55
**Compound 7**	0.006 µmol/mL	68.81 ± 4.76
**Compound 7**	0.06 µmol/mL	75.71 ± 13.39
**Compound 7**	0.3 µmol/mL	71.61 ± 0.94
**Compound 7**	1.0 µmol/mL	76.61 ± 7.10

The values given in bold represent statistically significant ($p < 0.05$) changes versus respective controls. The results are presented as mean ± SD; n = 4.

Supplementary Table S4. The release of tissue activator plasminogen (t-PA) from HUVEC cells in the presence of biguanides.

Compounds	Concentration	t-PA concentration [pg/mL]
Control		2297.8 ± 89.4
**Metformin**	0.006 µmol/mL	**2766.3 ± 136.3*** **
**Metformin**	0.06 µmol/mL	**2736.3 ± 74.6** **
**Metformin**	0.3 µmol/mL	**2652.3 ± 105.3*** **
**Metformin**	1.5 µmol/mL	**2623.2 ± 96.1* **
**Phenformin**	0.006 µmol/mL	2318.4 ± 224.3
**Phenformin**	0.06 µmol/mL	2204.5 ± 87.1
**Phenformin**	0.3 µmol/mL	2187.6 ± 159.3
**Compound 2**	0.006 µmol/mL	2399.7 ± 123.1
**Compound 2**	0.06 µmol/mL	1989.7 ± 202.9
**Compound 2**	0.3 µmol/mL	**448.4 ± 131.3*** **
**Compound 3**	0.006 µmol/mL	2438.9 ± 153.5
**Compound 3**	0.06 µmol/mL	**1642.8 ± 426.44* **
**Compound 3**	0.3 µmol/mL	**1374.1 ± 365.7* **
**Compound 3**	1.5 µmol/mL	**176.3 ± 69.7*** **
**Compound 8**	0.006 µmol/mL	2532.8 ± 115.4
**Compound 8**	0.06 µmol/mL	2592.7 ± 129.4
**Compound 8**	0.3 µmol/mL	**1573.2 ± 123.5** **
**Compound 8**	1.0 µmol/mL	**238.4 ± 25.8*** **
Control		*2356.5* ± 70.5
**Compound 4**	0.006 µmol/mL	2435.2 ± 30.5
**Compound 4**	0.06 µmol/mL	2236.8 ± 69.6
**Compound 4**	0.3 µmol/mL	**1559.6 ± 49.4*** **
**Compound 4**	1.0 µmol/mL	**271.2 ± 51.8*** **
**Compound 5**	0.006 µmol/mL	2361.8 ± 124.61
**Compound 5**	0.06 µmol/mL	2298.1 ± 46.0
**Compound 5**	0.3 µmol/mL	**1328.7 ± 18.5*** **
**Compound 5**	1.0 µmol/mL	**330.5 ± 96.7*** **
**Compound 6**	0.006 µmol/mL	2295.6 ± 135.9
**Compound 6**	0.06 µmol/mL	**1844.9 ± 36.7** **
**Compound 6**	0.3 µmol/mL	**399.0 ± 57.5*** **
**Compound 7**	0.006 µmol/mL	2289.3 ± 56.1
**Compound 7**	0.06 µmol/mL	2074.0 ± 219.3
**Compound 7**	0.3 µmol/mL	**1346.5 ± 211.9** **
**Compound 7**	1.0 µmol/mL	**947.1 ± 124.8*** **

The values given in bold represent statistically significant (* p < 0.05; ** p < 0.01; *** p < 0.001) changes versus respective controls. The results are presented as mean ± SD; n = 4-8.

Supplementary Table S5. The effects of biguanides on the expression of CD54 (ICAM-1) on the surface of endothelial cells.

Compound	Conc. [µmol/mL]	Gate B [%][1]	Gate C [%][2]
**Control I**	-	59.7 ± 2.6	26.7 ± 2.1
**Metformin**	0.06	62.4 ± 3.9	32.0 ± 7.3
	0.3	**65.9 ± 5.1***	33.1 ± 3.7
**Phenformin**	0.3	**72.3 ± 6.1****	27.3 ± 3.9
	1.0	**73.0 ± 8.3***	27.6 ± 2.3
**Comp. 8**	0.3	59.1 ± 1.5	25.8 ± 0.7
	1.0	**71.4 ± 1.2***	**68.3 ± 0.8*****
**Control II**	-	64.4 ± 2.0	27.6 ± 2.7
**Comp. 3**	0.06	67.6 ± 0.4	**41.1 ± 0.5***
	0.3	**84.9 ± 0.9****	**86.1 ± 0.8*****
**Comp. 5**	0.3	60.0 ± 6.8	**38.5 ± 1.6***
	1.0	63.7 ± 2.7	**46.9 ± 2.7***
**Control III**	-	67.6 ± 6.3	26.4 ± 2.4
**Comp. 2**	0.3	67.7 ± 2.3	**80.9 ± 1.2*****
	0.6	**40.2 ± 11.1****	**62.7 ± 11.7****
**Control IV**	-	63.9 ± 1.8	31.7 ± 3.0
**Comp. 4**	0.3	**78.6 ± 1.6*****	43.2 ± 2.0
	1.0	**85.3 ± 0.9*****	**62.4 ± 2.8****
**Comp. 6**	0.06	65.5 ± 2.8	**48.4 ± 1.8***
	0.3	63.4 ± 8.1	**55.0 ± 3.5****
**Comp. 7**	0.3	**65.0 ± 2.2***	24.6 ± 4.1
	1.0	**70.0 ± 2.1****	41.8 ± 4.52

Endothelial cells were treated with various concentrations of biguanides for 24 hours followed by staining with PE-CD54 antibody. The concentration for the studies were chosen on the basis of results collected in viability and integrity test. [1] – Cells gathered within the gate B reflect the % of acquired events existing as single cells (detection using FSC-A/FSC-H plots); [2] – the cells gathered in gate B were stained with CD54. The results are presented as mean ± standard deviation (SD), n = 3-6. The values given in bold represent statistically significant (*p < 0.05; ** p < 0.01; *** p < 0.001) changes versus respective controls.

Supplementary Table S6. The effects of biguanides on platelet-dependent thrombus formation using T-TAS system.

Compounds	Concentration	T_{10} [s]	OT [s]	CT [s]	AUC
Control	-	*133.7 ± 29.3*	*286.7 ± 65.5*	*153.0 ± 36.6*	*410.9 ± 52.2*
**Metformin**	0.06 µmol/mL	147.7 ± 49.5	370.0 ± 74.5	**222.3 ± 26.5***	362.4 ± 105.2
**Metformin**	0.3 µmol/mL	136.3 ± 25.1	**346.0 ± 47.0***	**209.7 ± 31.8***	367.3 ± 78.0
**Phenformin**	0.06 µmol/mL	168.7 ± 50.2	342.7 ± 105.7	174.0 ± 61.7	367.3 ± 76.1
**Phenformin**	0.3 µmol/mL	147.7 ± 17.0	330.0 ± 113.6	182.3 ± 97.7	377.1± 41.9
**Compound 2**	0.06 µmol/mL	114.0 ± 16.1	**354.7 ± 45.0***	**240.7 ± 43.5***	392.0 ± 26.4
**Compound 2**	0.3 µmol/mL	203.3 ± 61.8	**600.0 ± 1.0****	**396.7 ± 61.8***	**205.7 ± 51.3***
**Compound 3**	0.06 µmol/mL	155.3 ± 44.2	**370.3 ± 77.0***	**215.0 ± 34.0****	**362.8 ± 70.2***
**Compound 3**	0.3 µmol/mL	**195.3 ± 9.2***	**600.3 ± 0.6****	**405.0 ± 8.7****	**164.7 ± 57.7***
Control	-	*134.3 ± 26.7*	*285.3 ± 64.3*	*151.0 ± 43.6*	*407.2 ± 39.4*
**Compound 4**	0.3 µmol/mL	**171.3 ± 29.5***	432.0 ± 85.7	260.7 ± 66.5	**321.7 ± 46.7***
**Compound 4**	1.0 µmol/mL	**245.0 ± 34.4***	**600.7 ± 1.2****	**355.7 ± 35.5***	**117.2 ± 3.5****
**Compound 5**	0.3 µmol/mL	244.0 ± 41.1	**567.0 ± 39.0***	**323.0 ± 12.5***	**196.4 ± 26.1***
**Compound 5**	1.0 µmol/mL	95.7 ± 4.0	**425.7 ± 40.5***	**330.0 ± 38.2****	368.0 ± 26.5
**Compound 6**	0.06 µmol/mL	155.7 ± 57.5	372.7 ± 102.8	217.0 ± 87.7	371.6 ± 92.2
**Compound 6**	0.3 µmol/mL	182.7 ± 25.0	**527.0 ± 93.8***	344.3 ± 103.1	**293.0 ± 46.2****
**Compound 7**	0.3 µmol/mL	**177.7 ± 30.7***	388.7 ± 115.5	211.0 ± 86.3	329.5 ± 83.0
**Compound 7**	1.0 µmol/mL	**312.3 ± 81.0***	**600.3 ± 0.6****	**288.0 ± 80.9***	**103.6 ± 45.5***
**Compound 8**	0.3 µmol/mL	147.3 ± 27.5	376.0 ± 78.4	228.7 ± 66.5	369.8 ± 66.8
**Compound 8**	1.0 µmol/mL	**275.0 ± 86.3***	**594.0 ± 10.4****	**319.0 ± 82.6***	**144.3 ± 65.5***

The blood samples were incubated for 15 minutes with indicated concentrations of biguanide derivatives, and subsequently analysed by T-TAS system (PL-Chips). The results are presented as mean ± standard deviation (SD), n = 3. The values given in bold represent statistically significant ($p < 0.05$) changes versus respective controls. T_{10} – the onset of platelet thrombus formation, counted as the time for the flow pressure to increase to 10 kPa from baseline due to a partial occlusion of the capillary; OT - occlusion time, the time for the flow pressure to increase by 60 kPa from baseline owing to near complete occlusion of the capillary; CT - Clotting time – the difference between OT and T_{10}; AUC_{10} - area under the flow pressure curve for 10 minutes.

Supplementary Figure S3. Individual data for fibrin rich platelet thrombus formation under flow (T-TAS) by metformin and phenformin.

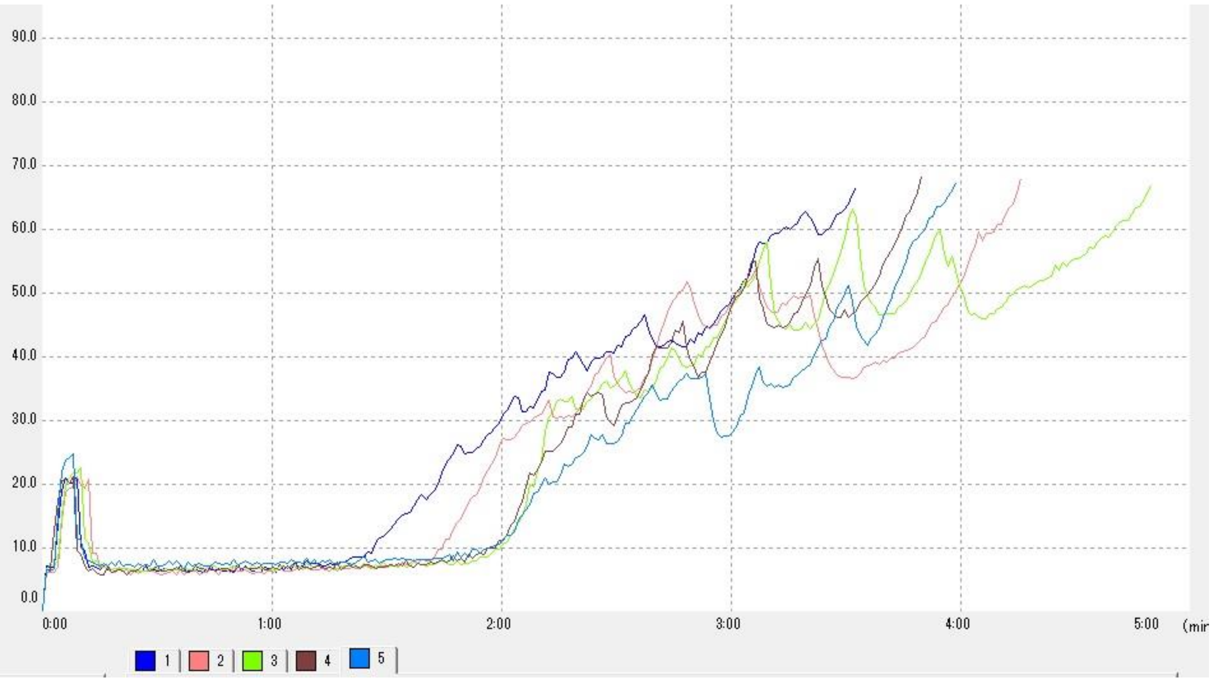

Representative plots for T-TAS (AR-Chips) data obtained in healthy control sample (navy blue colour) and after stimulation with metformin (0.06 μmol/mL – light pink, 0.3 μmol/mL - green) or phenformin (0.06 μmol/mL – brown, 0.3 μmol/mL – blue line).

Supplementary Table S7. The effects of biguanides on fibrin-rich platelet thrombus formation using T-TAS system.

Compounds	Concentration	T_{10} [s]	OT_{80} [s]	$T_{10–80}$ [s]	AUC_{30}
Control	-	*301.7 ± 21.2*	*414.7 ± 10.0*	*113.0 ± 13.0*	*1936.6 ± 18.8*
**Metformin**	0.06 μmol/mL	339.0 ± 6.6	439.7 ± 27.8	100.7 ± 21.4	1889.7 ± 28.6
**Metformin**	0.3 μmol/mL	348.0 ± 24.3	463.0 ± 22.1	115.0 ± 14.8	1870.0 ± 39.0
**Compound 3**	0.06 μmol/mL	363.3 ± 15.5	**486.3 ± 3.1****	123.0 ± 18.3	**1847.6 ± 12.3***
**Compound 3**	0.3 μmol/mL	402.7 ± 60.6	**537.3 ± 85.7***	134.7 ± 28.7	**1764.8 ± 106.5***
**Compound 6**	0.06 μmol/mL	**366.0 ± 26.2****	**480.7 ± 19.4****	114.7 ± 22.8	**1844.2 ± 24.1****
**Compound 6**	0.3 μmol/mL	**360.7 ± 25.8***	508.0 ± 60.0	147.3 ± 35.3	**1822.6 ± 63.8***
**Compound 8**	0.3 μmol/mL	306.3 ± 24.8	472.3 ± 97.4	166.0 ± 72.6	1863.8 ± 78.2
**Compound 8**	1.0 μmol/mL	**497.7 ± 31.5***	**606.0 ± 25.2****	108.3 ± 56.6	**1655.8 ± 16.6*****

The blood samples were incubated for 15 minutes with indicated concentrations of biguanide derivatives, and subsequently analysed by T-TAS system (AR-chips). The results are presented as mean ± standard deviation (SD), n = 3. The values given in bold represent statistically significant (*$p < 0.05$, ** $p < 0.01$, *** $p < 0.001$) changes versus respective controls. T_{10} – the onset of white thrombus formation; OT_{80} - complete occlusion of capillary due to thrombus formation; $T_{10–80}$ is the interval between T_{10} and OT; AUC_{30} - area under the flow pressure curve for 30 minutes.

Table S8. The summary of the results concerning the effects of metformin derivatives on selected parameters of vascular and plasma haemostasis.

**Compound**	**Integrity of HUVECs**	**Apoptosis**	**TF production in HUVECs**	**vWF release from HUVECs**	**t-PA release from HUVECs**	**ICAM-1 expression on HUVECs**	**Platelet thrombus formation**	**Blood coagulation**
Metformin	- (NS ↑)	↓	↑	-	↑	-	↑ OT, CT	-
Phenformin	- (‡)	↓	-	-	-	-	-	NE
1	↓	-	-	NE	NE	NE	NE	NE
2	- (‡)	↑	-	- (#)	↓	↑	↑ OT, CT, ↓ AUC	NE
3	-	↓	↑	- (#)	↓	↑	↑ OT, CT, ↓ AUC	↑ OT, ↓ AUC
4	- (‡)	↓	↑	-	↓	↑	↑ T_{10}, OT, CT, ↓ AUC	NE
5	- (‡)	↓	0	- (#)	↓	↑	↑ OT, CT, ↓ AUC	NE
6	- (‡)	-	0	-	↓	↑	↑ OT, CT	↑ T_{10}, OT_{80}, ↓ AUC
7	- (‡)	↓	0	-	↓	↑	↑ T_{10}, OT, CT, ↓ AUC	NE
8	- (‡)	- (‡)	0	↓	↓	-	↑ T_{10}, OT, CT, ↓ AUC	↑ T_{10}, OT_{80}, ↓ AUC

Abbreviations: NS – not significant; - no effects; - (‡) no effects apart from the highest concentration (1.5 µM/mL) for which decrease in integrity was reported; - (#) no effects apart from the highest concentration for which significant increase in vWF release was reported; ↑ or ↓ - significant increase or decrease in the respective effect over the entire or most of the concentrations tested; NE – not estimated; OT – Oclussion Time, CT – clotting Time, AUC – Area Under the Curve, T_{10} – the onset of platelets thrombus formation, OT_{80} - complete occlusion of capillary due to thrombus formation.

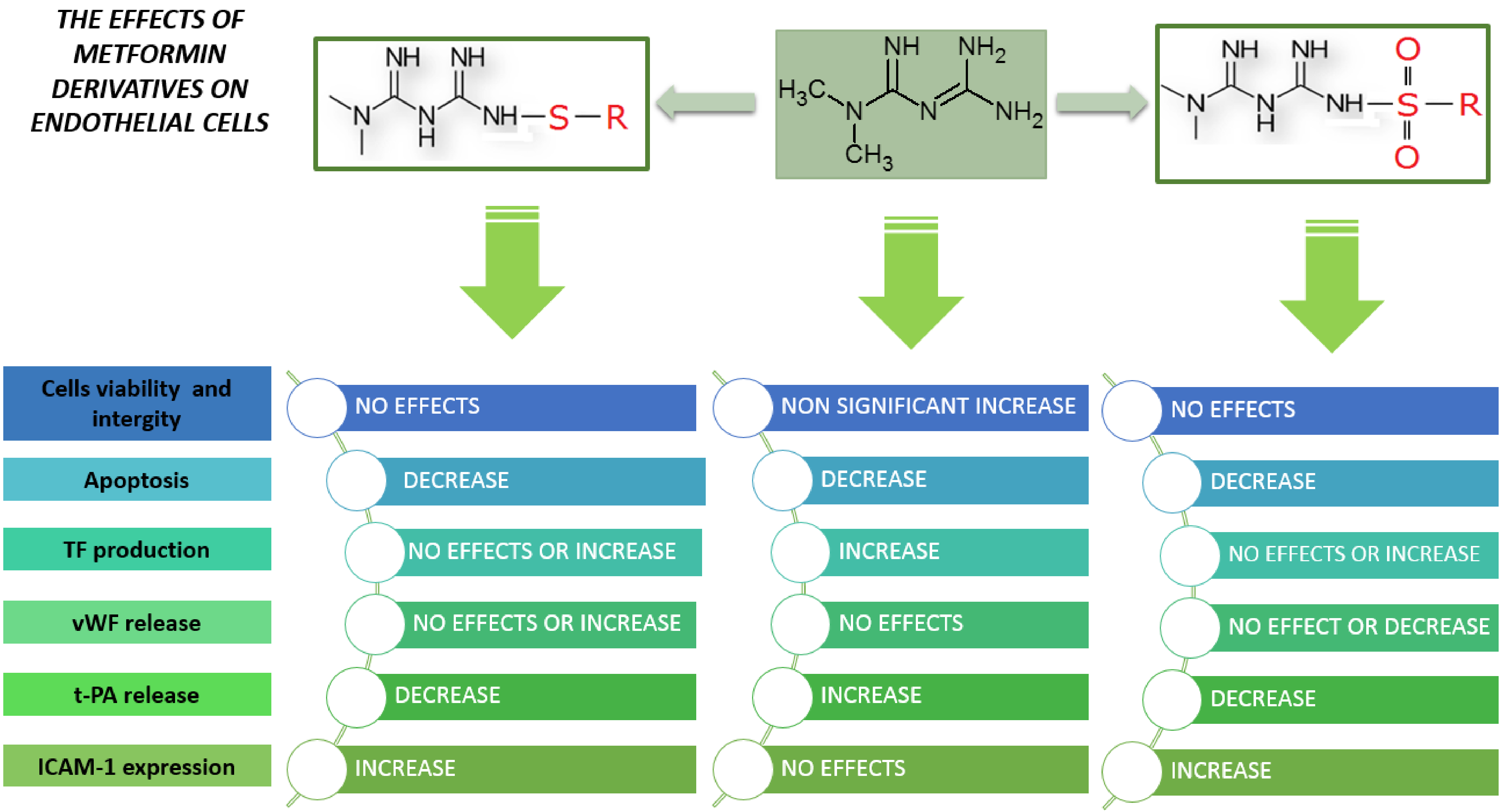

Figure S4. The summary effects of sulfenamide and sulfonamide derivatives of metformin on selected parameters of endothelial function.

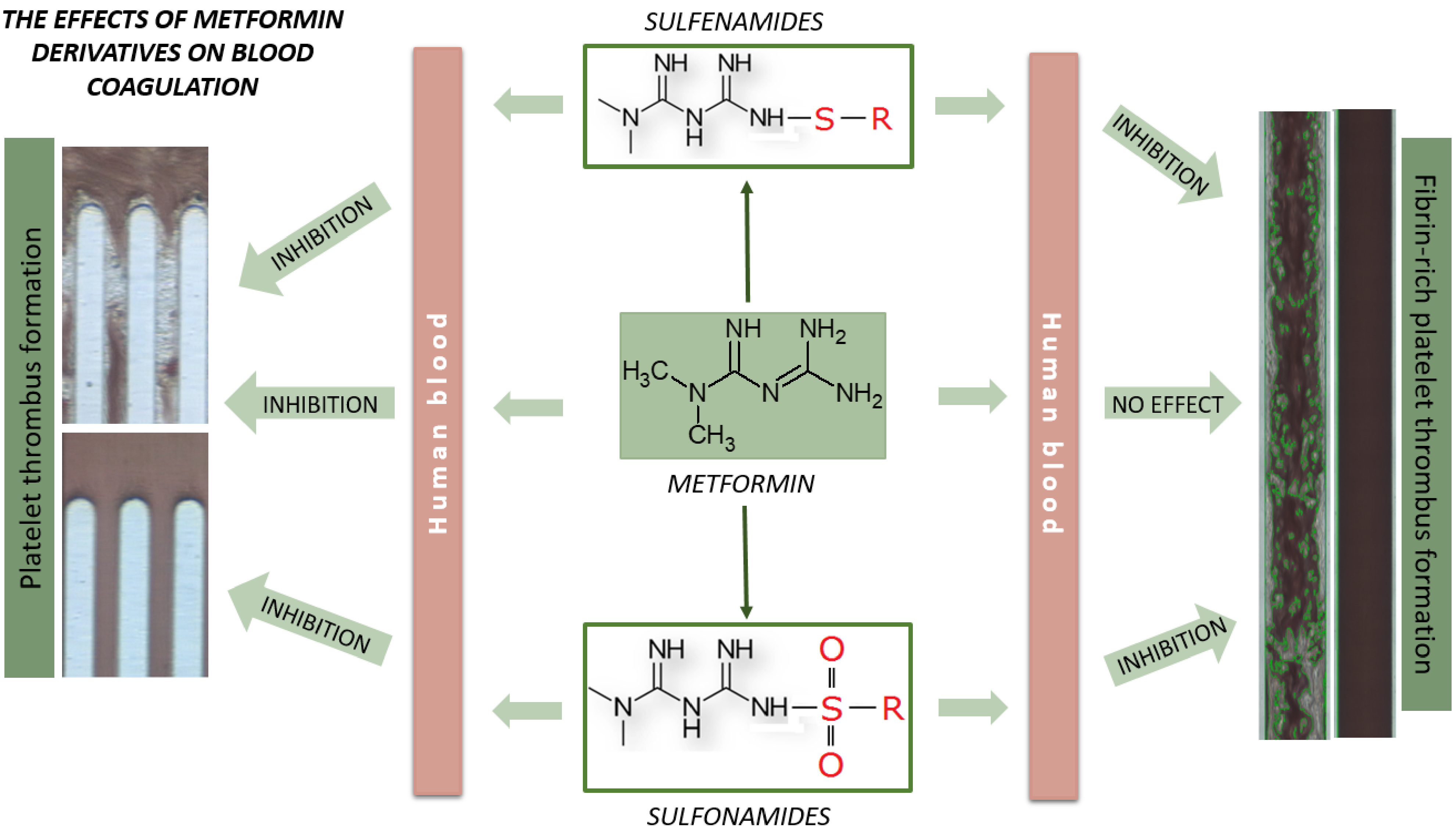

Figure S5. The summary effects of sulfenamide and sulfonamide derivatives of metformin on blood coagulation in semi-physiological conditions.

THE EFFECTS OF METFORMIN, COMPOUND 3 AND 8 ON SELECTED PARAMETERS OF VASCULAR AND PLASMA HAEMOSTASIS

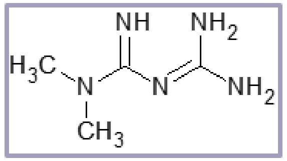

METFORMIN

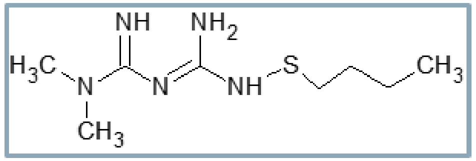

COMPOUND 3

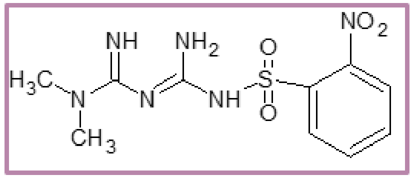

COMPOUND 8

	METFORMIN	COMPOUND 3	COMPOUND 8
**Cells viability and integrity**	Non significant increase	No effects	Decrease at 1.5 µmol/mL
**Apoptosis**	Decrease	No effects	Decrease at 1.5 µmol/mL
**TF production**	Increase	Increase	No effects
**vWF release**	No effects	Increase	Decrease
**t-PA release**	Increase	Decrease	Decrease
**ICAM-1 expression**	No effects	Increase	Increase at 1.0 µmol/mL
**Platelet thrombus formation**	Inhibition	Inhibition	Inhibition
**Fibrin-rich platelet thrombus formation**	No effects	Inhibition	Inhibition

Figure S6. The effects of *n*-butyl-sulfenamide and *o*-nitro-sulfonamide on the parameters of endothelial function and blood coagulation.
